# Antibiotic-persistent bacterial cells exhibiting low-level ROS are eradicated by ROS-independent membrane disruption

**DOI:** 10.1128/mbio.01199-25

**Published:** 2025-06-30

**Authors:** Yanghui Ye, Yuanqing Tian, Mingxin Duan, Weiwei Zhu, Jingyun Wu, Yilin Chen, Feng Xu, Xilin Zhao, Karl Drlica, Yuzhi Hong

**Affiliations:** 1 MOE Key Laboratory of Geriatric Diseases and Immunology, Suzhou Key Laboratory of Pathogen Bioscience and Anti-infective Medicine, Institute of Molecular Enzymology, School of Life Sciences, Suzhou Medical College, Soochow University 12582, Suzhou, Jiangsu, China; 2 State Key Laboratory of Vaccines for Infectious Diseases, Xiang-An Biomedicine Laboratory, National Innovation Platform for Industry-Education Integration in Vaccine Research, School of Public Health, Xiamen University 12466, Xiamen, Fujian, China; 3 Jiangsu Provincial Medical Innovation Center of Trauma Medicine, Key Laboratory of Alkene-Carbon Fiber-Based Technology & Application for Detection of Major Infectious Diseases, Department of Emergency Medicine, Institute of Trauma Medicine, The First Affiliated Hospital of Soochow University, Suzhou, China; 4 Public Health Research Institute and Department of Microbiology, Biochemistry & Molecular Genetics, New Jersey Medical School, Rutgers Biomedical and Health Sciences, Rutgers University, Newark, New Jersey, USA; Instituto de Biologia Molecular y Celular de Rosario, Rosario, Santa Fe, Argentina

**Keywords:** antibiotic, cell death, persistence, tolerance, reactive oxygen species, membrane damage

## Abstract

Bacterial persistence increases therapy duration, disease relapse, and antibiotic resistance. Mechanisms underlying persistence and feasible ways to rapidly eliminate persister cells are largely unknown. The present work examined genetic and environmental perturbations to identify anti-death events occurring in *Escherichia coli* persister and phenotypically tolerant cells. The quiescent status of *hipA7* and *metG2* persister cells, which were protected from killing by multiple antibiotics, was insensitive to the presence/absence of exogenous nutrients. In contrast, stationary-phase and nutrient-starved wild-type cultures, which displayed tolerance rather than the subpopulation status of persistence, were readily killed by ciprofloxacin upon restoration of nutrients, thereby indicating that tolerance was phenotypic. Both persistent and tolerant cells suppressed accumulation of reactive oxygen species (ROS), DNA breakage, and global metabolic activity. Restoration of nutrients to stationary-phase cultures restored these three processes for phenotypically tolerant cells but not for persister cells. Cultures of high-frequency-persistent *hipA7* and *metG2* mutants and low-frequency-persistent wild-type cells were rapidly sterilized by ROS-independent, synergistic membrane disruption using aminoglycoside-polymyxin combinations; rapid eradication occurred at clinically achievable concentrations for both antibiotics. The aminoglycoside-polymyxin combination also killed environmentally tolerant cells but more slowly. The combination killed both laboratory and clinical isolates of gram-negative bacteria, an *E. coli ptsI* mutant that is pan-tolerant to diverse antibiotics and disinfectants, and *E. coli* cells in a biofilm model. Moderate lethality was observed with the gram-positive bacterium *Staphylococcus aureus*. The work indicates that suppression of ROS accumulation is a common feature of persistence and phenotypic tolerance, and it emphasizes ROS-independent strategies for controlling quiescent bacterial populations.

**IMPORTANCE:**

The report generalizes the concept that persistence and tolerance involve suppression of toxic metabolites (reactive oxygen species [ROS]). The work also shows that an environmental perturbation (nutrient deprivation) leads to antibiotic tolerance rather than persistence, thereby raising questions about the classification of other environmental perturbations. The synergistic action of multiple aminoglycoside species with polymyxins opens many treatment options. Lethality with biofilms and with *S. aureus* may extend polymyxin-based therapies beyond planktonic, gram-negative bacteria, and the ROS independence of the combination may allow antioxidant mitigation of drug toxicity. Overall, the work advances our knowledge of persistent and tolerant bacterial pathogens and our efforts to eradicate them.

## INTRODUCTION

Globally, bacterial infections have reached a crisis state due to an increasing prevalence of antibiotic failure and bleak prospects for finding new antibiotic classes (1). Failure falls into two general categories: resistance (absence of growth inhibition by antibiotics) and persistence/tolerance (absence or delay of killing by antibiotics with no effect on growth inhibition) (2–6). The distinction between resistance and persistence/tolerance fits with antibiotic lethality being a two-step process in which the first step is the formation of bacteriostatic, drug-target lesions (blocked by resistance mechanisms) (7), while the second step is either (i) conversion of the bacteriostatic lesion into a lethal one, such as conversion of DNA complexed with fluoroquinolone and gyrase into fragmented chromosomes (8) that are not repaired, or (ii) a toxic metabolic response to the lesions (9) that is blocked by persistence/tolerance mechanisms (6). The metabolic response is commonly thought to involve increases in reactive oxygen species (ROS) (6, 9–14). Each step is clinically important: resistance allows bacterial growth during infection, while persistence and tolerance restrict antibiotic lethality, lengthen treatment time, and contribute to both disease relapse (15–18) and the emergence of resistant mutants (19, 20).

While resistance has been extensively studied, much less is known about persistence and tolerance. Tolerance has been attributed to growth defects (21) that would interfere with the lethal metabolic response to antibiotics; with rapidly growing cells, tolerance interferes with lethal pathways that involve the accumulation of ROS (6, 22). Persistence and tolerance are distinguished by tolerance referring to survival of the bulk population, which displays a gradual drop in survival (2, 5, 21, 23, 24), while persistence refers to a subpopulation for which survival displays an initial rapid drop followed by a quasi-plateau (5). Whether persistence involves suppression of ROS accumulation is unknown. Indeed, how persister cells survive bactericidal antibiotics is poorly understood, as are clinically feasible strategies for rapidly eradicating them.

Persistence is likely present in all bacterial populations as low-level survival to antibiotic-mediated killing, but it can be dramatically elevated via mutation. An early example is the *hipA7* allele of the HipAB toxin-antitoxin system (21, 25–27). This mutation raises bacterial survival to β-lactams and fluoroquinolones by several orders of magnitude in a process that involves the stringent response and elevated (p)ppGpp production (26, 28). The relative activity of the HipA toxin and the cognate HipB antitoxin likely accounts for maintenance of the subpopulation status characteristic of persistence. Another example is the *metG2* persistence mutation, which also raises survival by several logs through elevation of (p)ppGpp (29). Environmental perturbations, such as nutrient deprivation (suspension of cells in saline or growth to stationary phase), have also been considered to be a form of persistence (30–32). Comparison of environmental and genetic forms of persistence is expected to reveal common features and thereby help explain the suppression of antibiotic lethality.

The present work began by examining the ability of antibiotics to kill *Escherichia coli* grown to stationary phase or starved of nutrients. These environmental perturbations blocked killing by a variety of antibiotics: complete survival indicated antibiotic tolerance rather than persistence. Extensive killing when nutrients were added led to the tolerance being termed phenotypic. One feature of persistent mutants was the inability of nutrients to render them vulnerable to antibiotic lethality. Another was suppression of antibiotic-induced ROS, a property shared with phenotypic tolerance and probably with other forms of persistence and tolerance. ROS suppression and vulnerability to ROS-independent killing directed efforts to control persister cells toward strategies that would bypass protective anti-oxidant defenses. Membrane-damaging agents were attractive, especially combinations of two agents having different action mechanisms such that each would enhance the lethal effects of the other. An ROS-independent combination of polymyxin and aminoglycoside met expectations as it sterilized cultures of wild-type, *hipA7,* and *metG2* cells at clinically attainable concentrations within a few hours.

## RESULTS

### Phenotypic tolerance associated with nutrient depletion

**Fig 1:**
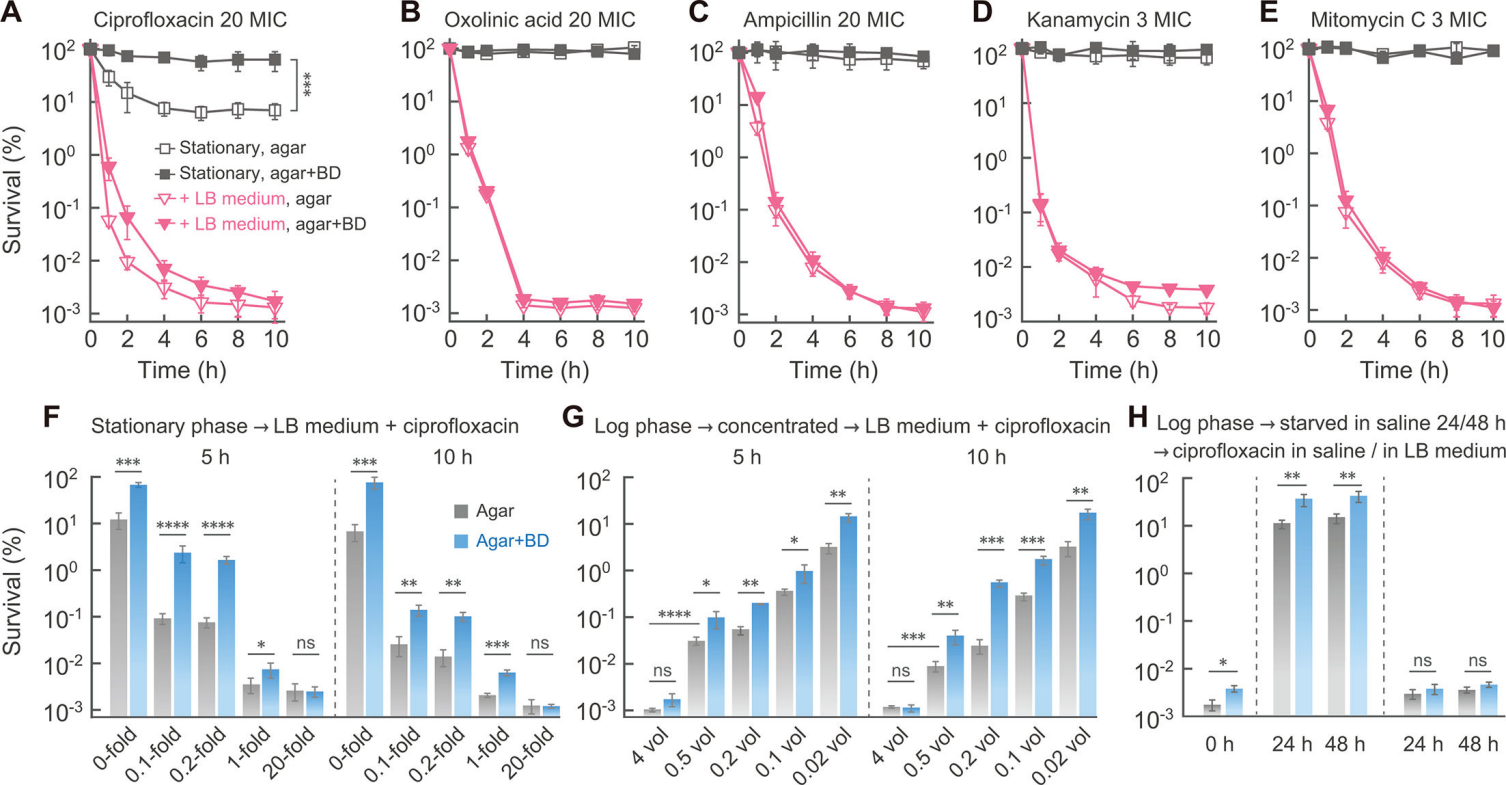
Antibiotic lethality with phenotypically tolerant cells. (A−E) Nutrient restoration enables killing of stationary-phase, phenotypically tolerant cells by several antimicrobial classes. Stationary-phase cultures of wild-type *E. coli* (strain 0001) were treated with antibiotic as indicated in the absence or presence of a 20-fold dilution into fresh Luria-Bertani (LB) medium (*n* = 5 with each experiment). BD, bipyridyl plus DMSO in agar. (**F**) Extent of dilution correlates with extent of tolerant survival. Stationary-phase cultures (*n* = 3) were treated with ciprofloxacin for 5 or 10 h during the addition of 0.1, 0.2, 1, or 20 volumes of fresh LB medium, indicated by fold dilution. (**G**) Cell density correlates with tolerant survival to antimicrobial. Log-phase cells (OD_600_ = 0.3, *n* = 3) were collected by centrifugation, resuspended in the indicated volumes of fresh LB medium, and immediately treated with ciprofloxacin. (**H**) Starvation-mediated tolerance reversed by nutrients. Log-phase cells (OD_600_ = 0.3, *n* = 5) were washed twice with saline, resuspended in saline, and incubated for 24 or 48 h. The cells were then treated with ciprofloxacin for another 5 h and plated on agar either lacking or containing BD. Aliquots of the starved cells were also resuspended in fresh LB medium containing ciprofloxacin for another 5 h. The antibiotic concentrations were selected to ensure rapid killing of susceptible cells while minimizing intracellular drug residue following washing. In all panels, *n* is the number of replicate experiments; they showed similar results. Data represent the mean ± SD. *, *P* < 0.05; **, *P* < 0.01; ***, *P* < 0.001; ****, *P* < 0.0001; ns, no significance.

We examined two environmental models considered to reflect persistence, culture growth to stationary phase, and nutrient starvation for effects on antibiotic-mediated killing of *E. coli*. Bacterial cultures were grown to stationary phase, treated with the fluoroquinolone ciprofloxacin at 20 MIC, and tested for survival with the standard agar assay. As reported previously (27, 32–34), survival was 10%, roughly 10,000 times higher than observed with exponentially growing cells (Fig. 1A; Fig. S1). The apparent subpopulation behavior of ciprofloxacin was not due to incubation time, as similar results were obtained with cultures in stationary phase for 12 and 24 h (Fig. S2A). However, when ciprofloxacin was replaced with oxolinic acid, a first-generation quinolone whose members kill only by an ROS-dependent mode (35), survival was 100% (Fig. 1B). Full survival of stationary-phase cultures was also seen with three other antibiotic classes represented by ampicillin, kanamycin, and mitomycin C (Fig. 1C through E). Since the survival of a subpopulation is central to the definition of persistence (5), complete blockage of killing by members of four different antibiotic classes forces us to conclude that stationary-phase-mediated protection from antimicrobial lethality is a form of tolerance rather than persistence. Interestingly, when subinhibitory concentrations of anti-oxidants (bipyridyl plus dimethyl sulfoxide [DMSO]) were present in agar used to assay survival, complete survival was observed for ciprofloxacin-treated stationary-phase cultures (Fig. 1A). In the case of ciprofloxacin, the apparent subpopulation behavior was due to ROS-dependent death after removal of ciprofloxacin rather than persistence. Presumably, non-lethal DNA damage occurring during stationary phase (36) was carried over to drug-free agar, where it stimulated a lethal ROS response (13).

We expected stationary-phase-mediated protection from killing to be a phenotypic phenomenon (3) associated with nutrient deprivation. As a test, we restored nutrients to stationary-phase cultures by dilution of cells into fresh medium containing lethal concentrations of antibiotics. Restoration of nutrients allowed extensive killing by each of several antibiotics to the same low level (0.001%; Fig. 1A through E). In these five cases, the addition of anti-oxidants to assay agar failed to affect survival, indicating that either cell death occurred before plating or that ROS are not involved in post-stress death under these conditions. Since ciprofloxacin is widely used in studies of persistence and tolerance, we focused subsequent work on this quinolone. For this work, we chose to use the optimal bactericidal level (20 MIC; Fig. S1A) because ultrahigh concentrations lead to the Eagle effect (37), to an ROS-independent mode of killing (38), and, in the present work, to erosion of the protective effect of persistence mutations (Fig. S3D, H, and L).

When we varied diluent volume and presumably nutrient level, intermediate levels of survival were observed at intermediate levels of dilution (survival ranged between 0.01% and 0.001% when equal or greater volumes of fresh medium plus ciprofloxacin were added; Fig. 1F). Moreover, a positive relationship between survival and cell density was seen when log-phase cells were resuspended in fresh, ciprofloxacin-containing medium: resuspension in 0.02- to 4-fold volumes of medium resulted in survival ranging from 5% to 0.001% (Fig. 1G). In this case, dense cultures were expected to be nutrient limited. Similar results were obtained following dilution of cells starved for nutrients by incubation in saline (Fig. 1H). Collectively, the data indicate that the protective effect of nutrient limitation on antibiotic lethality is readily reversible; this phenotypic tolerance is distinct from genetic tolerance with rapidly growing cells (6, 22) and persistence resulting from mutations, such as *hipA7* and *metG2,* as described below.

### Persistence and nutrient restoration

**Fig 2:**
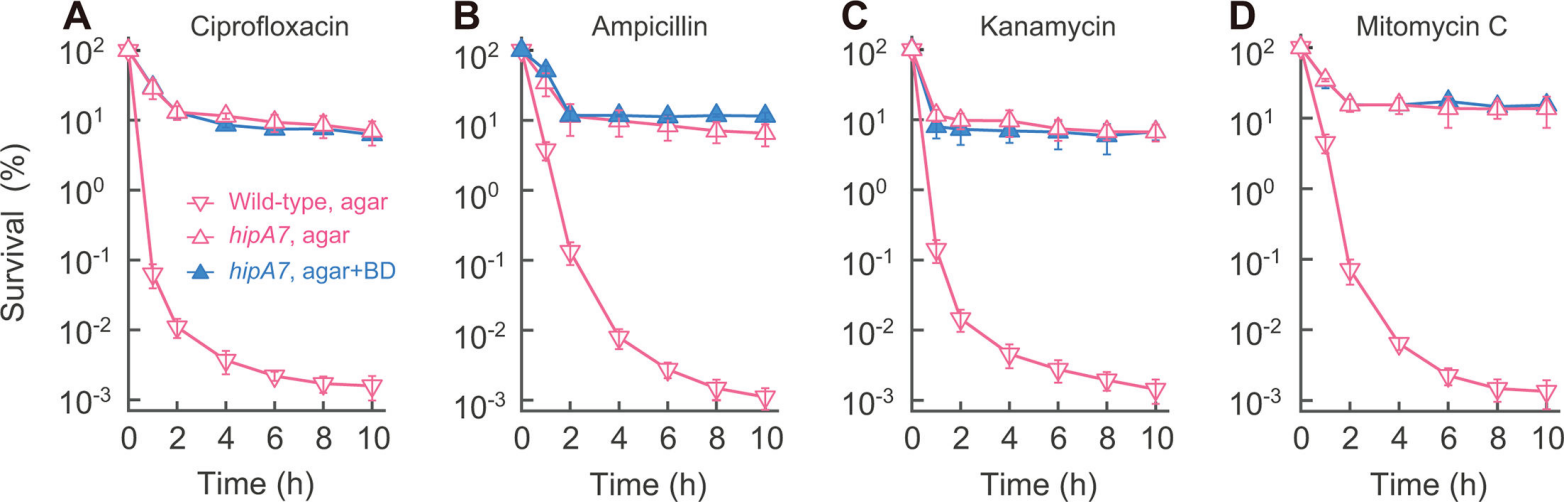
Persistent survival to four bactericidal antibiotic classes. (A−D) Nutrient restoration lacks an effect on persistent survival. Stationary-phase *hipA7* cultures (strain 0022) were diluted 20-fold in fresh medium containing 20 MIC ciprofloxacin, 20 MIC ampicillin, 3 MIC kanamycin, or 3 MIC mitomycin C (*n* = 3 for each experiment). CFU was determined on both LB agar and agar containing bipyridyl plus DMSO (BD). In all panels, *n* is the number of replicate experiments; similar results were seen in replicates. Data represent the mean ± SD.

Since a small fraction (0.001%) of wild-type cells persisted when treated with four antibiotic classes in rich medium (Fig. 1A through E), we asked whether high-level persistence also allows restoration of killing when nutrients are added. Members of four antibiotic classes failed to extensively kill stationary-phase cultures of *hipA7* and *metG2* mutants, as seen with wild-type cells (Fig. S4). But unlike wild-type cultures, survival remained at 10% after a 20-fold dilution of stationary-phase *hipA7* cultures into fresh, ciprofloxacin-containing medium (Fig. 2A; anti-oxidants in agar provided little protection), which was about 10,000-fold higher than wild-type persistence (Fig. 1A). A similar persistence frequency (~10%) was seen when *hipA7* cultures were treated with lethal concentrations of ampicillin, kanamycin, or mitomycin C following dilution into fresh medium (Fig. 2B through D). Nutrient-independent, high-frequency persistence also occurred with an *metG2* mutant: survival remained at 1%–5% (Fig. S5). Thus, *hipA7* and *metG2* mutations confer high-level survival (5%–10%) to four antibiotic classes with no additional killing during a shift to rich medium (Fig. 2; Fig. S5). These data demonstrate a qualitative difference between persistence and phenotypic tolerance.

### Suppression of ROS level by persistence

**Fig 3:**
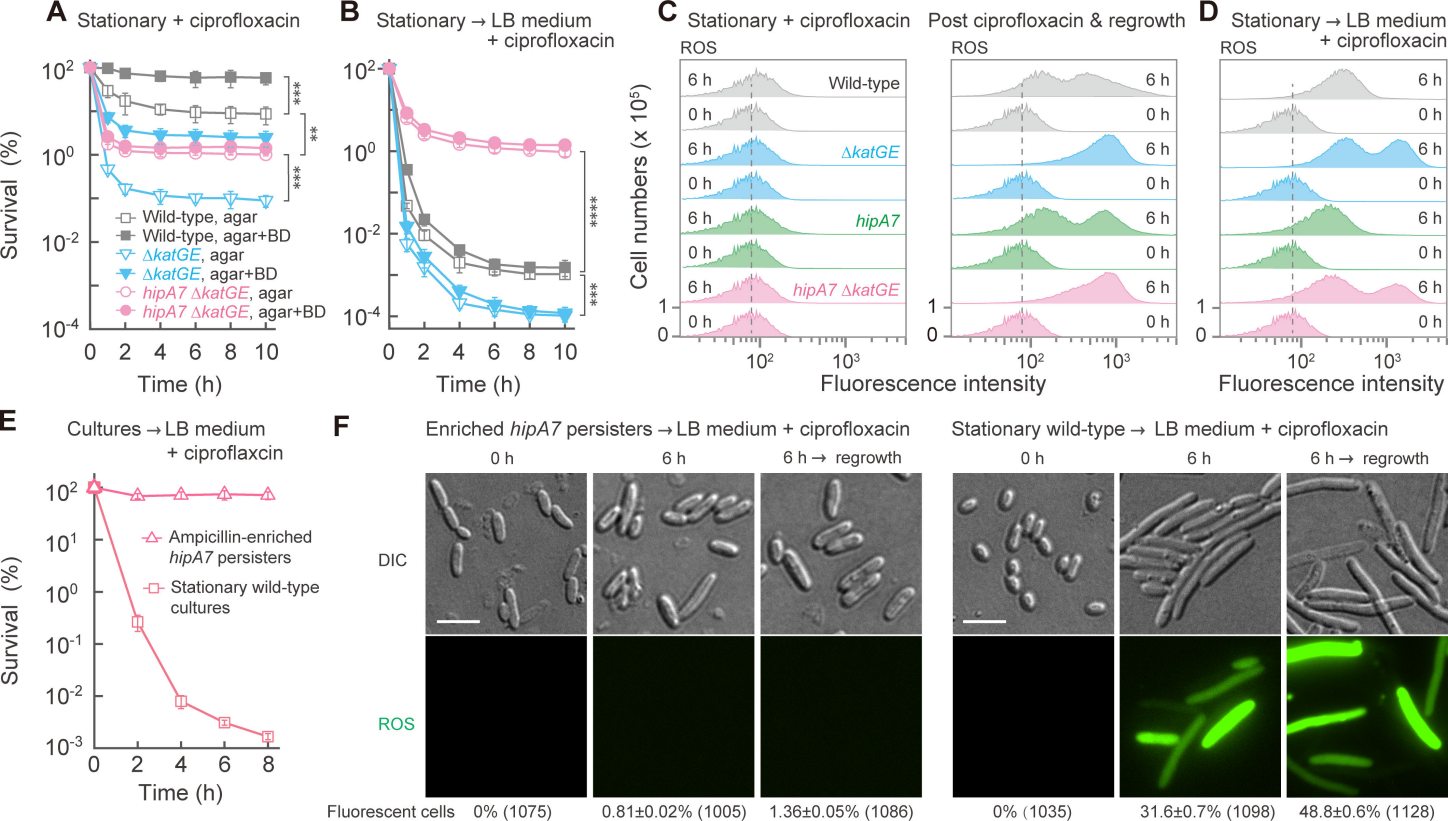
Suppression of ROS levels associated with phenotypic tolerance and *hipA7* persistence. (**A**) Deficiency of catalases increases killing of stationary-phase, tolerant cultures. Stationary-phase cultures of the ∆*katGE* (strain 0042), *hipA7* ∆*katGE* (strain 0354), and wild type (strain 0001) were treated with 20 MIC ciprofloxacin (*n* = 4). Survival was determined by plating on LB agar and on agar containing bipyridyl plus DMSO (BD). (**B**) Little effect of catalase deficiency on *hipA7* persistence. Strains and experimental conditions as in panel A, except that cultures were diluted 20-fold in fresh medium during treatment (*n* = 4). (**C**) Fluorescence indicating intracellular ROS of stationary-phase cells during and after antibiotic treatment. Left: indicated cultures (*n* = 3) were treated with ciprofloxacin as in panel A in the presence of carboxy-H2DCFDA. Right: stationary-phase cultures, treated with ciprofloxacin without carboxy-H2DCFDA, were washed and then incubated in antibiotic-free, fresh medium (for regrowth) containing the ROS indicator carboxy-H2DCFDA for 3 h. Fluorescence was measured by flow cytometry. (**D**) ROS levels of stationary-phase cultures treated with ciprofloxacin when diluted into fresh medium. Strains and treatment (*n* = 3) were as panel C, except fresh medium was included during treatment as in panel B. (**E**) Survival of enriched persisters treated with ciprofloxacin. Persisters of *hipA7* (strain 0022) were enriched by ampicillin treatment for 10 h in LB medium, washed, and then treated with 10 MIC ciprofloxacin for 8 h in fresh medium. Wild-type cultures, diluted into fresh medium containing ciprofloxacin, served as a control. (**F**) ROS-mediated fluorescence determined at the single-cell level. *hipA7* persisters (*n* = 3) were enriched and treated as in panel E, except carboxy-H2DCFDA was present during treatment. For panels labeled “regrowth,” cells were treated with ciprofloxacin in the absence of carboxy-H2DCFDA, washed to remove ciprofloxacin, and then incubated in drug-free, fresh medium containing the ROS indicator for 3 h to reveal post-antibiotic ROS accumulation. DIC, differential interference contrast. Bar = 5 µm. In panels A, B, and E, the values are presented as the mean ± SD. In panels C, D*,* and F, one of three independent biological replicates was shown as a representative.

Since ROS is commonly thought to contribute to the lethal action of antibiotics (9, 12, 39), we examined the possibility that phenotypic tolerance and persistence suppress ROS accumulation. To test this idea, we deleted catalase genes (∆*katGE*), which was expected to raise intracellular hydrogen peroxide levels (6, 10, 12, 40–42). Following ciprofloxacin treatment of stationary-phase cultures, catalase mutant survival was about 100-fold lower than observed with wild-type, catalase-containing cells (Fig. 3A). Survival increased 20-fold when assay agar contained anti-oxidants (Fig. 3A), as expected for peroxide being involved in death occurring after ciprofloxacin removal. Dilution of the ∆*katGE* mutant into fresh medium containing ciprofloxacin dropped survival frequency to 0.0001%, which was about 10-fold lower than observed with wild-type cultures (Fig. 3B and 1A). In this case, the addition of anti-oxidants to the assay agar had no effect, presumably because killing was rapid and extensive during the drug treatment period. Deletion of *katGE* in the *hipA7* mutant also reduced the fraction of surviving cells in cultures, from about 10% to 1% (Fig. 3A and B). These data indicate that ROS participate in killing the major portion of cultures.

Several additional experiments characterized the catalase deficiency effects. In one, placement of *katE* in the low-copy plasmid pACYC184 restored survival of the catalase-deficient mutant to wild-type levels (Fig. S6). This complementation experiment supported the conclusion that reduced survival was due to the absence of catalase. We also reversed the effect of the catalase deficiency by adding anti-oxidants (bipyridyl plus DMSO) to stationary-phase cultures of Δ*katGE* cells (Fig. S7). Since *E. coli* has a third enzyme, alkyl hydroperoxide reductase (AhpCF) that could affect ROS levels, we examined an *ahpCF* deficiency. We found little effect on survival to ciprofloxacin treatment (Fig. S8). Thus, catalase appears to dominate hydrogen peroxide removal during antibiotic treatment of *E. coli*.

We next monitored intracellular ROS using the dye carboxy-H2DCFDA, which fluoresces when oxidized (12). Stationary-phase cultures showed little ROS accumulation arising from ciprofloxacin treatment (Fig. 3C; Fig. S9A), although survival measurements showed differences among the wild-type, ∆*katGE*, *hipA7*, and *hipA7* ∆*katGE* strains (Fig. 3A and 2A). The survival assay appears to be more sensitive to a catalase deficiency than the ROS test. Nevertheless, shifting stationary-phase cells to nutrient-rich medium resulted in a surge in ROS that revealed the ROS elevation expected for the catalase-deficient mutants (Fig. 3D; a measurable *hipA7* effect on ROS was not expected in this experiment because the mutation would affect only 10% of the population). As expected, a suspension of wild-type, log-phase cells in nutrient-deficient saline showed little ROS signal when treated with ciprofloxacin (Fig. S9C). Also expected was plasmid-mediated complementation of catalase defects by ROS measurements (Fig. S6C). Collectively, these data indicate that protection from ciprofloxacin-mediated killing involves suppression of ROS accumulation.

When we treated cultures with ciprofloxacin after shifting stationary-phase cultures to fresh medium, ROS levels rose, with the Δ*katGE* mutant exhibiting a peak of exceptionally high ROS level (Fig. 3D). With this mutant, an ROS peak similar to that seen with wild-type cells appeared at early times and was followed later by the second, high-level ROS peak (Fig. S9B). We speculate that the high-level peak arose from multiple rounds of DNA damage producing ROS that in turn caused more DNA damage (13) through an iterative process that is poorly controlled in the absence of catalase.

As pointed out, the subpopulation nature of persistence allowed *hipA7* cultures to show ROS changes similar to those of cells having a wild-type *hipA* allele, although with a slightly lower level of ROS (Fig. S9B). To assess persister ROS level, we enriched the persister subpopulation by treatment of *hipA7* cultures with ampicillin for 10 h followed by washing (ampicillin was expected to lyse non-persister cells [21]). Enrichment was 10-fold, to approximately 100% of the population. The enriched *hipA7* persister cells were not killed by ciprofloxacin (Fig. 3E; Fig. S10), and little ROS signal was seen among the persister cells (0.8% and 1.4% positive cells) during treatment with ciprofloxacin in fresh medium and during regrowth after antibiotic removal, respectively (left panel in Fig. 3F; in these preparations, cell lysis by ampicillin eliminated non-persister cells). The absence of extensive DNA damage due to ciprofloxacin was also apparent by the lack of cell filamentation characteristic of ciprofloxacin-treated wild-type cells. In contrast, bulk, wild-type cells displayed a strong ROS signal (32% and 49% positive cells) during and after treatment, respectively (right panel in Fig. 3F). The ampicillin-enriched persisters exhibited no growth lag when diluted into fresh medium, and growth rates were comparable to wild-type cultures (Fig. S11A). Overall, the data indicate that ROS accumulation and ciprofloxacin-mediated killing are suppressed during persistence.

### Suppression of DNA breakage and translation in persister cells

**Fig 4:**
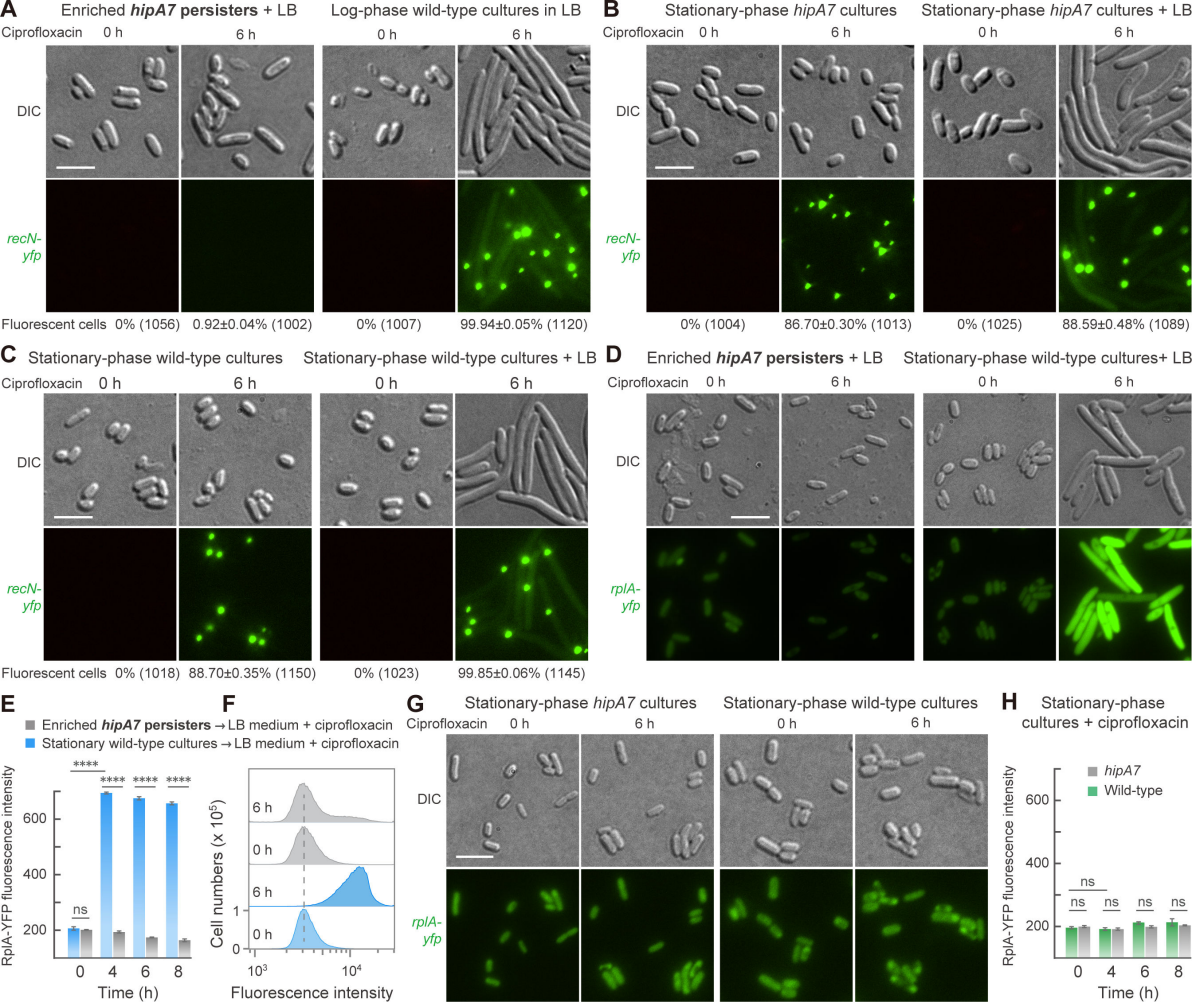
Low levels of fluoroquinolone-mediated DNA breakage and translation in persister cells. (**A**) Little DNA breakage. Persister cells of the *hipA7::recN-yfp* strain (0140, *n* = 3), enriched by ampicillin, were treated with 10 MIC ciprofloxacin for 6 h in fresh medium. As a positive control for DNA breaks, log-phase wild-type cultures (strain 0141, *n* = 3) were treated with ciprofloxacin. (**B, C**) DNA breakage in bulk cultures. Stationary-phase cultures of the *hipA7* mutant (strain 0140) and wild-type (strain 0141) strains were treated with ciprofloxacin for 6 h; parallel cultures were treated after a 20-fold dilution into fresh medium with ciprofloxacin (*n* = 4). (**D, E**) Translational activity indicated by RplA-YFP fluorescence. The *hipA7::rplA-yfp* strain (strain 0355, *n* = 3) was treated as in panel A. As a control, stationary-phase cultures of wild-type cells, containing *rplA-yfp* (strain 0182, *n* = 3), were treated with ciprofloxacin after a 20-fold dilution into fresh medium. The mean values of RplA-YFP fluorescence in the imaged cells at the indicated treatment time are expressed in relative units. (**F**) Suppressed expression of RplA-YFP in persister cells. Strains and conditions were as in panel D. Fluorescence was assayed by flow cytometry. (**G, H**) Suppressed expression of RplA-YFP during stationary phase. Stationary-phase cultures of the strains in panel D were directly incubated with ciprofloxacin (*n* = 3). The mean values of RplA-YFP fluorescence in cells are expressed in relative units. In all panels except F, cells were imaged by fluorescence microscopy; bar indicates 5 µm. DIC, differential interference contrast. Parentheses show the total number of cells counted in images of three independent experiments. ****, *P* < 0.0001; ns, no significance.

Growing cells treated with ciprofloxacin contain DNA breaks (8, 43). Since breaks likely induce ROS (13) and also result from ROS action (11, 44), we expected persisters to exhibit fewer ciprofloxacin-mediated DNA breaks than wild-type cells. To test this idea, we constructed strains containing a chromosomal *recN-yfp* element that reports double-stranded DNA breaks as fluorescence (41, 45) (the *recN-yfp* element had little effect on growth following dilution from stationary phase; Fig. S11B). At 10 MIC ciprofloxacin, almost 100% of log-phase, wild-type cells were fluorescent after a 6 h treatment (Fig. 4A). We then enriched *hipA7* persister cells using ampicillin and treated them with ciprofloxacin (6 h). Although persister cells were capable of forming colonies, only a small fraction (about 1%) of the enriched cells exhibited fluorescent foci (Fig. 4A; Fig. S12A; ampicillin-mediated lysis eliminated dead cells). Thus, few persistent *hipA7* cells contain detectable DNA breaks. We also examined DNA breakage with cultures that had not been enriched for persisters. When stationary-phase *hipA7* cultures were treated with ciprofloxacin, the fraction with fluorescent foci was 87% (Fig. 4B), indicating that the bulk population was subject to DNA damage. After dilution of stationary-phase *hipA7* cells into rich medium containing ciprofloxacin, which has little effect on the survival of the *hipA7* mutant (Fig. S12B), the fraction of cells containing fluorescent foci was largely unchanged (89%; Fig. 4B). These data are consistent with a persistent fraction (10% of the population) remaining unaffected by the addition of nutrients (Fig. 2A).

A wild-type culture behaved differently. Without anti-oxidant addition, the death of wild-type, stationary-phase cells was ~90%, consistent with 89% of cells showing fluorescent foci that indicate DNA-damaged cells (Fig. 1A and 4C; Fig. S12D). When these stationary-phase cells were diluted into rich medium, which also contained ciprofloxacin, the fraction showing DNA breakage increased to 99.9% (Fig. 4C; Fig. S12E), and almost all cells were killed (0.001% survival; Fig. S12A). Addition of nutrients likely allowed the accumulation of toxic metabolites, including ROS, that increased the DNA damage (11). Thus, stationary-phase wild-type cells exhibit an increase in DNA-damaged cells upon dilution that is not seen with the *hipA7-*persistent subpopulation.

We also examined translation as a representative of global metabolism (46) using strains containing *yfp* fused downstream from the chromosomal gene *rplA*, which encodes the 50S ribosomal protein L1. As with observations of ROS, little increase in fluorescence was observed when stationary-phase, bulk cultures of the *hipA7* mutant were treated with ciprofloxacin (Fig. 4G and H). When *hipA7* persister cells were enriched by ampicillin treatment and treated with ciprofloxacin in rich medium, the fluorescent signal was lower than seen with wild-type, stationary-phase cultures shifted to ciprofloxacin-containing medium (Fig. 4D through F; Fig. S13). Collectively, the data indicate that *hipA7* persister cells, but not wild-type cells, maintain a quiescence state when shifted to rich medium in which ROS accumulation, DNA damage, and translation are suppressed.

### Eradication of persister cells by synergistic lethality of aminoglycoside-polymyxin combinations

**Fig 5:**
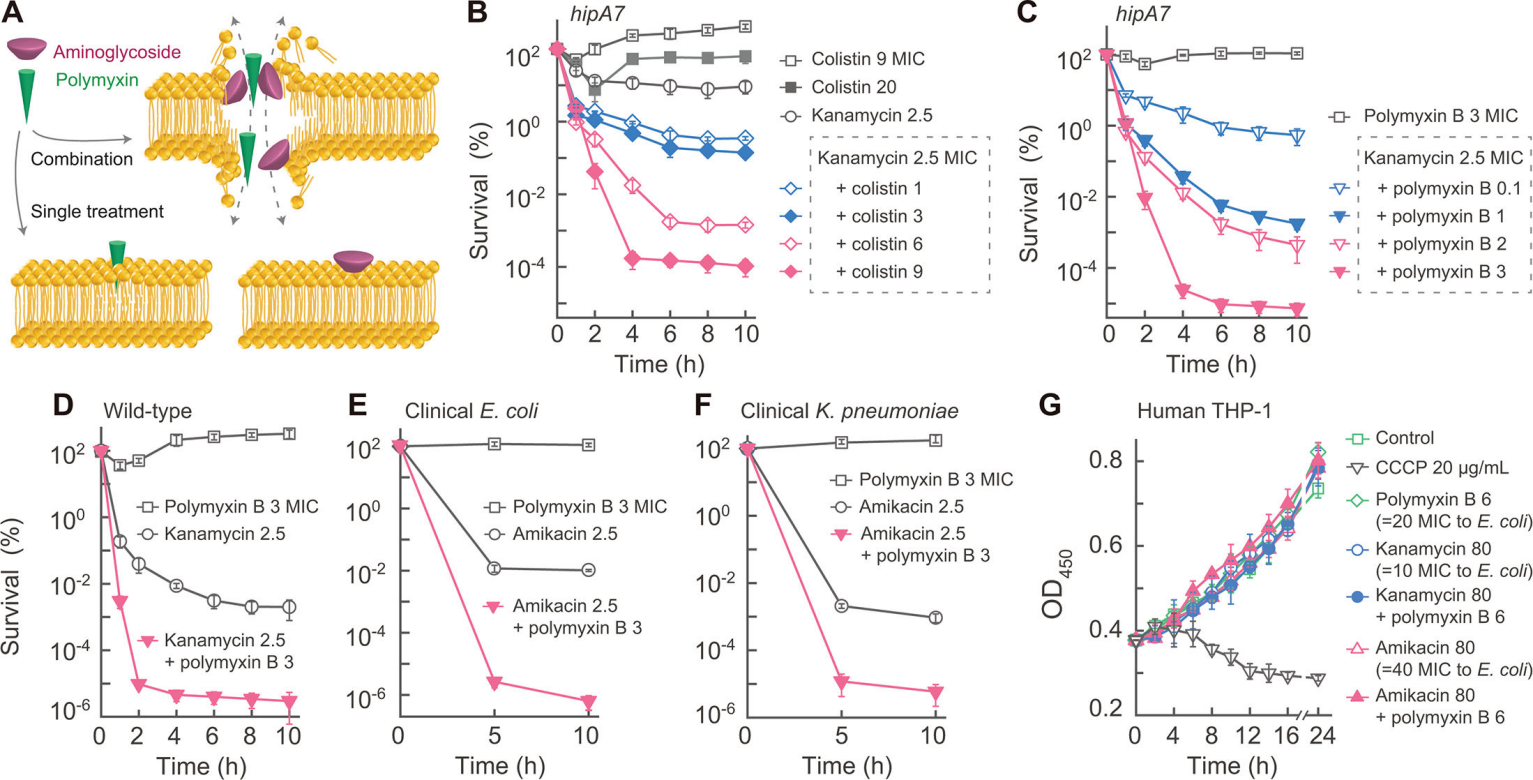
Elimination of persister cells by polymyxin-aminoglycoside combinations. (**A**) Schematic explanation of membrane disruption from cotreatment with aminoglycoside and polymyxin. (**B**) Killing of *hipA7* mutant by kanamycin, colistin, or their combinations. Stationary-phase *hipA7* cultures (strain 0022, *n* = 3) were diluted 20-fold into fresh LB medium containing kanamycin (2.5 MIC), colistin (9 or 20 MIC), or their combinations (all concentrations indicated in panel are times MIC). (**C**) Killing of *hipA7* mutant by kanamycin, polymyxin B, or their combinations. Strain and conditions (*n* = 3) are as in panel B, except polymyxin B was substituted for colistin. (**D**) Reduction of wild-type persistence level by a kanamycin-polymyxin combination. Wild-type cultures (strain 0001, *n* = 3) were treated as in panel B. (**E, F**) Effect of amikacin-polymyxin combination on killing clinical isolates of *E. coli* and *K. pneumoniae*. Cultures of *E. coli* isolate 0284 and *K. pneumoniae* isolate 0210 (*n* = 3, each) were prepared as in panel B and treated with amikacin at 2.5 MIC, polymyxin B at 3 MIC, or a combination of the two (concentrations shown in panels B–F are times MIC). (**G**) Effect of antibiotics on human THP-1 cell growth. THP-1 cells (*n* = 3) were treated with kanamycin, amikacin, polymyxin B, or their combinations as indicated. Carbonyl cyanide m-chlorophenylhydrazone (CCCP) served as a control (drug concentrations in panel G are in μg/mL). Growth was determined using a CCK-8 assay. Data represent the mean ± SD.

Since persister cells suppress processes that increase ROS levels, we reasoned that it would be difficult to kill persisters by attempts to artificially elevate ROS to lethal levels. We hypothesized that exceptional membrane-disrupting activity would kill persister cells in an ROS-independent way. To this end, we combined two agents having different membrane-perturbing mechanisms, expecting damage caused by one agent to facilitate damage by the other (Fig. 5A). Preliminary screening of such agents led to an aminoglycoside-polymyxin combination. In initial experiments, kanamycin was applied at its peak serum concentration attained during standard treatment of humans (20 µg/mL, 2.5 MIC) (47), and colistin (polymyxin E) was administered at 0.9 µg/mL (9 MIC), which was slightly below its maximal serum concentration (48). Testing involved survival measurement after shifting stationary-phase cultures to antibiotic-containing medium.

The two compounds showed little killing of a *hipA7* mutant when tested individually: colistin showed no killing; kanamycin reduced survival to about 10% (Fig. 5B). When the two compounds were applied as a combination, survival dropped a millionfold to 10^−4^%. When polymyxin B replaced colistin, survival decreased another 10-fold to 10^−5^% (Fig. 5C), essentially eradicating the bacterial population. Reducing the concentration of colistin in the combination by 2/3 (to 0.3 µg/mL, 3 MIC) raised survival a 1,000-fold (to 0.1%). Reducing polymyxin B concentration to 0.1 or 1 MIC raised *hipA7* survival to 1% or 0.001%, respectively, which was still 10- to 10,000-fold lower, respectively, than with kanamycin alone. Thus, the enhanced lethal effect of the combination depends on drug concentration. Likewise, the combination decreased the survival of wild-type cultures, which had a low frequency of persistence, from 0.001% to 10^−6^% or 1,000-fold lower than values seen with single-agent treatments (Fig. 5D and 1A through E).

As with kanamycin, combinations pairing polymyxin B with other aminoglycoside derivatives, such as amikacin, tobramycin, gentamicin, or streptomycin, exhibited exceptional persistence-eradicating lethality with wild-type strains and with *hipA7* and *metG2* mutant cultures (Fig. S14). When kanamycin was combined with ciprofloxacin or ampicillin, the persistence level of the *hipA7* mutant and the wild-type strain showed little change (Fig. S15B). Combining kanamycin with mitomycin C lowered survival of *hipA7* cultures by only 100-fold, and several combinations with ciprofloxacin or ampicillin showed at most a 10-fold effect (Fig. S16). Thus, the exceptional lethality of aminoglycoside-polymyxin synergy was not general to antibiotics. Our results fit with previous work that, unlike our study, used concentrations that are higher than used clinically (33, 49). Finding that clinically relevant concentrations are effective encouraged further characterization.

### Effects of aminoglycoside-polymyxin against diverse bacteria, biofilm, and cultured human cells

We performed several tests to determine whether the combination has broad-spectrum activity. One test focused on *E. coli* mutants (∆*ptsI*, ∆*cyaA*, and ∆*crp*) that are pan-tolerant to a variety of lethal antibiotics and disinfectants (6) and other mutants associated with tolerance: ∆*nhaA* (50), ∆*dnaK*, ∆*dnaJ*, ∆*dksA*, ∆*yigB,* and ∆*ihfA* (51, 52). When stationary-phase cultures of the mutants were diluted into fresh medium and treated for 5 h with kanamycin (2.5 MIC) plus polymyxin B (3 MIC), survival dropped seven orders of magnitude (Fig. S17). For phenotypically tolerant cultures grown to stationary phase, killing took longer, starting after 2 d. By 7 d of treatment, survival dropped five to seven orders of magnitude, which was 10^4^- to 10^6^-fold lower than single-drug treatments (Fig. S18).

Another test focused on a variety of wild-type strains. For example, the combination of aminoglycoside (1.25–2.5 MIC) plus polymyxin B (3 MIC) severely reduced persistent survival to 10^−5^%–10^−6^% with four clinical isolates of *E. coli* and three isolates of *Klebsiella pneumoniae* (Fig. 5E and F; Fig. S19A through F). The combination treatment also reduced by ~10-fold the persistence level found for aminoglycoside alone for laboratory strains of *Pseudomonas aeruginosa* and *Staphylococcus aureus*, lowering survival level to 0.001% (Fig. S19G and H). Thus, enhanced killing by an aminoglycoside-polymyxin combination applies to persister cells present in cultures of gram-negative and gram-positive (*S. aureus*) bacteria, although *E. coli* and *K. pneumoniae* are especially susceptible.

Since many infections involve biofilms that contain persister cells, we also examined the effect of an aminoglycoside-polymyxin combination with a biofilm model composed of either *E. coli* or *K. pneumoniae*. When biofilms, formed by static growth for 48 h, were treated with 2.5 MIC kanamycin plus 3 MIC polymyxin B, survival decreased by 10^4^- to 10^5^-fold (Fig. S20). In contrast, treatment with the individual agents exhibited no killing of biofilm cells. These results emphasize the potential importance of the combination.

We next asked whether the combination was generally toxic to human cells. When we exposed cultured human THP-1 macrophage-like cells to high concentrations of the drug combination (polymyxin B at 6 µg/mL, 20 MIC and amikacin or kanamycin at 80 µg/mL, 10 MIC) or the single agents for 24 h, no obvious growth inhibition occurred (Fig. 5G). These drug concentrations were 5- to 20-fold higher than required for sterilization of persistent bacterial cultures (Fig. 5). Additional work is needed to evaluate the potential toxicity of the combinations in animal models.

### Mechanism of synergistic lethality involves ROS-independent membrane disruption

The striking bactericidal effect of antibiotic cotreatment was associated with reduction in MIC by 10- to 16-fold for the kanamycin-polymyxin B combination and by 4- to 5-fold for the kanamycin-colistin combination relative to the MICs of the individual compounds. The fractional inhibitory concentration index for kanamycin, when combined with polymyxin B, was 0.19 and when combined with colistin was 0.44 (Fig. S21; index values ≤0.5 reflect a positive bacteriostatic interaction between two antibiotics [53]). A bacteriostatic interaction between the aminoglycosides and polymyxins would likely facilitate downstream lethal action.

To examine a role for proton motive force (PMF) in lethality, the PMF inhibitor carbonyl cyanide m-chlorophenylhydrazone (CCCP) was added during antibiotic treatment of *hipA7* cultures in rich medium. CCCP increased viability by 10^6^-fold for *hipA7* cells treated with kanamycin plus polymyxin B (Fig. S15D). These data are consistent with persister-cell PMF being crucial for the uptake of aminoglycoside and polymyxin (54–56).

We also examined possible ROS involvement in the lethality of aminoglycoside-polymyxin combinations. A deficiency of *katGE* failed to increase killing of the *hipA7* mutant or wild-type strain; moreover, the addition of anti-oxidants (bipyridyl plus DMSO) had little effect, both during treatment in liquid medium or on agar after antibiotic removal (Fig. S22). Thus, killing by combinations of aminoglycoside and polymyxin occurs in a largely ROS-independent manner.

**Fig 6:**
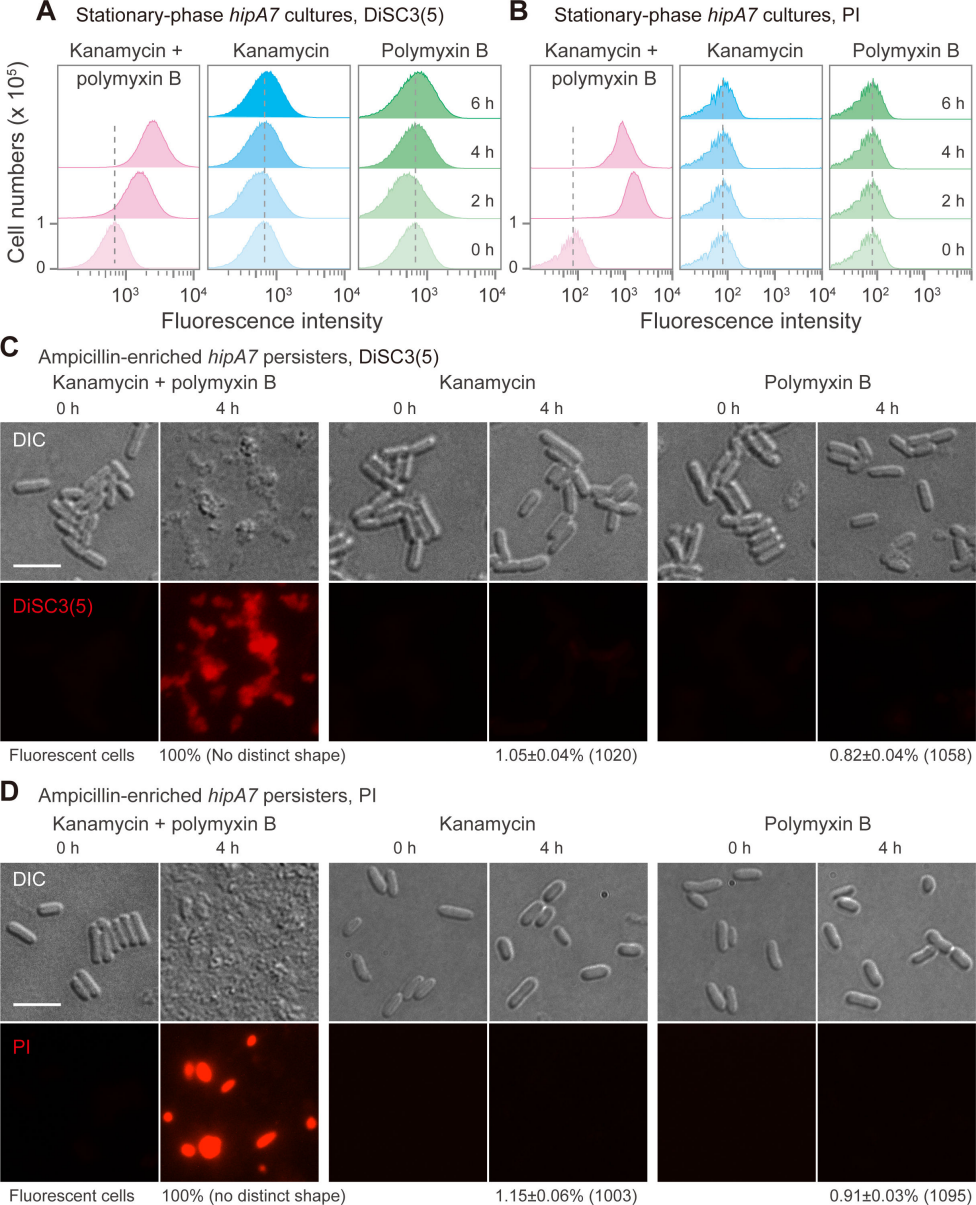
Membrane disruption due to cotreatment with polymyxin and aminoglycoside. (**A**) Effect of aminoglycoside-polymyxin combination on membrane potential indicated by DiSC3(5) fluorescence. Stationary-phase *hipA7* cultures (strain 0022) were diluted 20-fold into fresh LB medium containing 2.5 MIC kanamycin, 3 MIC polymyxin B, or the combination. Each culture also contained 2.5 µM DiSC3(5). Fluorescence of DiSC3(5) at the indicated times after dilution was detected by flow cytometry. (**B**) Effect of aminoglycoside-polymyxin combination on membrane permeability reported by propidium iodide (PI) fluorescence. *hipA7* cultures were prepared as in panel A except propidium iodide (5 µM) was substituted for DiSC3(5). (**C, D**) Membrane damage in enriched persisters due to antibiotic combination. Stationary-phase cultures of the *hipA7* mutant (*n* = 3) were diluted into fresh LB medium and treated with 20 MIC ampicillin for 10 h, washed, and then treated with 2.5 MIC kanamycin plus 3 MIC polymyxin for 4 h in fresh medium containing DiSC3(5) (**C**) or propidium iodide (**D**). Differential interference contrast (DIC). Bar = 5 µm. In all panels, three independent biological replicates produced similar results. Numbers in parentheses indicate the total number of cells counted.

To better understand how persister cells are killed by aminoglycoside-polymyxin cotreatment, cell membrane potential and permeability were examined using the fluorescent dyes DiSC3(5) and propidium iodide, respectively. Stationary-phase *hipA7* cultures were diluted into fresh medium containing DiSC3(5) and treated with kanamycin (2.5 MIC) plus polymyxin B (3 MIC). After 2–4 h of cotreatment, the DiSC3(5) signal increased two- to threefold, as determined by flow cytometry (Fig. 6A). In contrast, treatment with kanamycin or polymyxin B individually caused little increase in the DiSC3(5) signal (Fig. 6A). Thus, the reduction of membrane potential correlates with the death of persister cells arising during combination treatment.

When we labeled cells diluted into fresh medium with propidium iodide and treated them with kanamycin plus polymyxin B, the fraction of fluorescent *hipA7* mutant cells was 100%, as measured by flow cytometry and microscopy (Fig. 6B; Fig. S23A). Treatment with kanamycin or polymyxin B alone showed fluorescence in only 7%–8% of the population, consistent with the high-level survival determined by agar plating performed in parallel (Fig. 2A and 5B). Cotreatment also increased the fluorescent signal associated with propidium iodide-stained *metG2* mutant cells (Fig. S23).

When we enriched *hipA7* persisters by lysis of susceptible cells using ampicillin and then treated with a kanamycin-polymyxin combination, the persisters lost their distinct cell shape and gained fluorescent signals from DiSC3(5) and propidium iodide (Fig. 6C and D). These observations are consistent with membrane damage. In contrast, treatment with kanamycin or polymyxin B individually had little effect on cell shape and did not increase DiSC3(5) or propidium iodide signals from ampicillin-enriched persister cells (Fig. 6C and D). Collectively, the data indicate that a combination of polymyxin and aminoglycoside has a synergistic, membrane-disrupting ability that rapidly lowers survival of persistent cells in an ROS-independent manner.

## DISCUSSION

**Fig 7:**
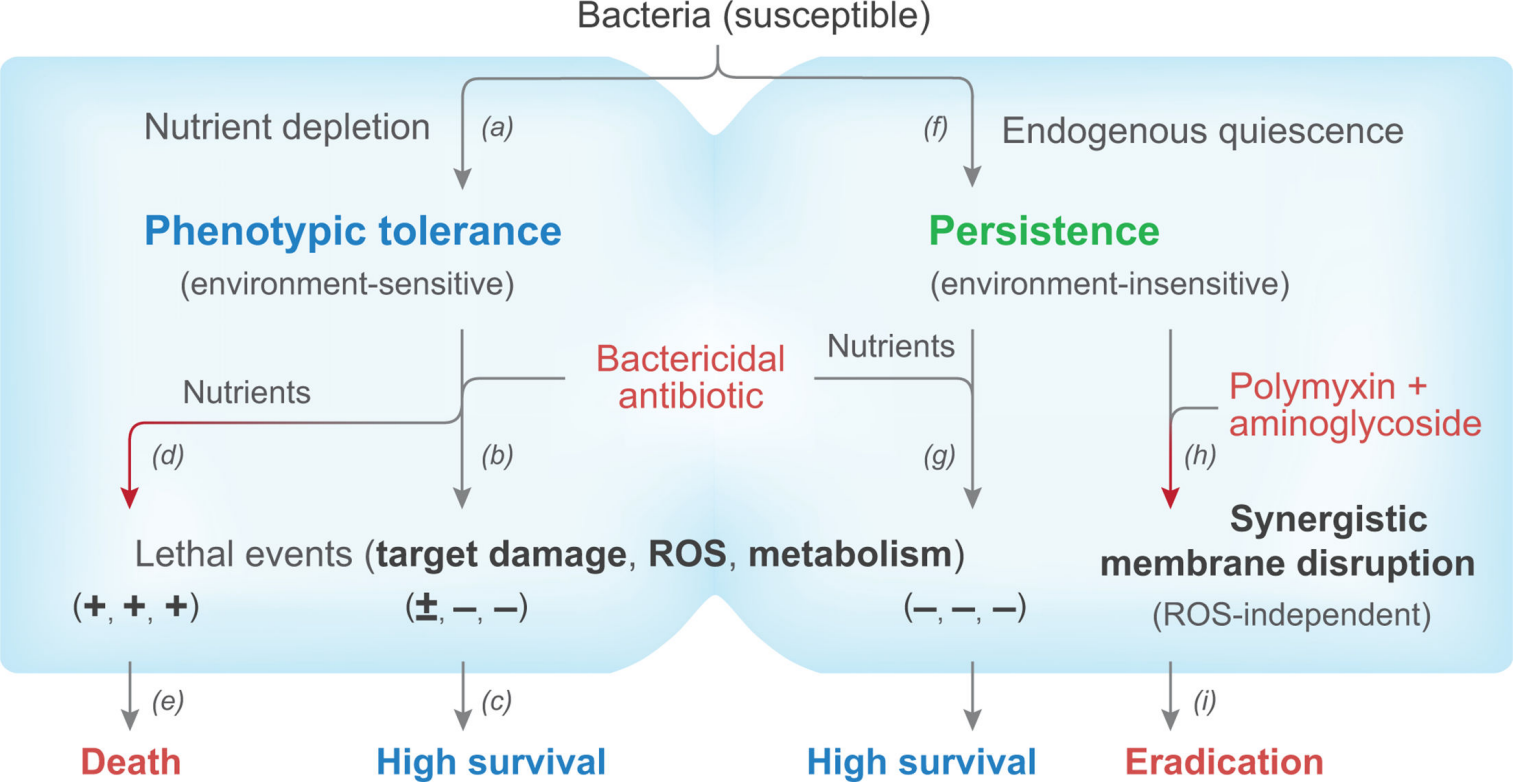
Distinction between phenotypic tolerance and persistence. (*a*) Nutrient depletion due to stationary phase or resuspension in saline produces phenotypic tolerance. (*b, c*) This tolerance protects the bulk population from lethal events, and cells survive bactericidal treatment. (*d, e*) Response to nutrients causes the cells to lose tolerance and die by ROS-associated processes that include drug target damage, ROS accumulation, and metabolic activity. (*f, g*) Persister cells as subpopulations in mutant (*hipA7, metG2*) and wild-type cultures survive treatment with a variety of bactericidal antibiotics, even in rich medium, due to endogenous quiescence. (*h, i*) Synergistic, ROS-independent membrane disruption by polymyxin-aminoglycoside combinations at clinically attainable concentrations leads to rapid eradication of pan-persistent cells and genetic pan-tolerant mutants.

Antimicrobial persistence and tolerance in bacteria, fungi, parasites, and mammalian (cancer) cells make control of disease difficult (2, 23, 24, 57). The present study advances our understanding of these two protective features in bacteria (sketched in Fig. 7). Two phenomena were examined: environmental situations likely to involve nutrient limitation and mutations that stimulate the stringent response. In both cases, *E. coli* cells suppressed ROS accumulation during treatment with the fluoroquinolone ciprofloxacin. That suggested that controlling persistent cells might best be achieved by bypassing potential problems associated with ROS suppression: use ROS-independent ways to kill bacteria. When we examined membrane damage generated by a combination of aminoglycoside and polymyxin, this ROS-independent approach eradicated persister cells at drug concentrations low enough for the strategy to be clinically feasible. Moreover, an ROS-independent approach is expected to stimulate work on antioxidant strategies for mitigating nephrotoxicity associated with polymyxin and aminoglycoside treatment. Overall, the work (i) suggests that other forms of persistence and tolerance also involve suppression of ROS and (ii) emphasizes the special nature of membrane-damaging antibiotic combinations for addressing problems of persistence and tolerance.

Conclusions about suppression of ROS can be derived from (i) increased killing of a catalase-deficient mutant by ciprofloxacin, (ii) decreased killing when the drug was combined with anti-oxidants, and (iii) the expected changes in fluorescence of a dye known to be sensitive to radicals. These examples, which included wild-type persistence that was lowered by the absence of catalase, add to two cases of genetic tolerance in rapidly growing cells (6, 22) and to data indicating that many antibiotics kill bacteria by increasing intracellular ROS (9–13, 41, 58, 59). These observations make it likely that growth- or metabolic-defect forms of antibiotic persistence and tolerance (5, 21, 60) are also characterized by low ROS levels. Suppression of ROS level is also associated with the Eagle effect when observed with the quinolone nalidixic acid (37). In this phenomenon, bacteria fail to be killed by very high drug concentrations in a Lon protease-dependent manner (35). Since Eagle effect survival can be 100% (37, 61), the effect is likely to be a form of tolerance. We expect future work to show that suppression of ROS accumulation is a general protective feature of bacteria during antibiotic and disinfectant treatment.

Persister cells, enriched from *hipA7* cultures, exhibited two ancillary properties that fit with low ROS values being protective. One is suppression of quinolone-mediated DNA breakage, which is thought to arise in part from ROS action (chloramphenicol blocks ROS accumulation and chromosome fragmentation with first-generation quinolones [37, 43]). When we probed for ciprofloxacin-mediated breakage with RecN-YFP, we detected more cells with breakage in the bulk population than in the persister subpopulation. The second property is low translation activity, which is consistent with suppression of metabolism and protection from antimicrobial lethality (62, 63). Slow metabolism, which would impede the generation of ROS, is likely a consequence of HipA7 stimulating the stringent response and induction of (p)ppGpp (26, 28). Elevation of (p)ppGpp levels, which has been observed with many examples of persistence and tolerance (64, 65), likely impedes the cAMP-Crp-ROS death pathway stimulated by antibiotics and disinfectants (6, 22).

Environment- and mutation-mediated protection differed in several ways that led us to question whether environmental models reflect persistence. The central aspect of persistence is its subpopulation status (5). Finding that growth to stationary phase protected 100% of an *E. coli* culture from oxolinic acid, ampicillin, kanamycin, and mitomycin C suggested tolerance. Although the finding of 10% survival to 20 MIC ciprofloxacin supported protection of a subpopulation, as previously reported (32, 33), complexity is expected from residual lethal action on assay agar after removal of ciprofloxacin and other fluoroquinolones (13, 66, 67). Addition of anti-oxidants to agar brought survival with ciprofloxacin to 100% with stationary-phase cells. Our interpretation is that DNA damage occurs during this phase (36); during plating on agar, damage repair induces too much ROS for cells to suppress. That allows anti-oxidants added to the agar to increase survival.

Another difference between the two types of protection involves the addition of nutrients to stationary-phase cultures along with ciprofloxacin. Wild-type cells are rapidly killed, while the *hipA7* mutant exhibits a drug concentration effect. At 20 MIC ciprofloxacin, 10% survival was observed; raising the concentration to 100 or 500 MIC lowered survival to 0.2%, a drop of 50-fold. One interpretation is that the *hipA7* mutation is unable to protect from ROS-independent chromosome fragmentation occurring at very high fluoroquinolone concentration. This complexity argues against the common practice of using ultra-high antibiotic concentrations to study the metabolic response of persister cells. An additional concern about using high concentrations of antibiotics, such as aminoglycosides, is that the drug can be difficult to remove prior to plating, thereby confounding survival measurements (13).

ROS suppression and endogenous quiescence inherent to persistence caused us to seek a membrane-disrupting strategy that would bypass ROS defenses, thereby sterilizing persister cultures. Pilot experiments using a variety of membrane-perturbing agents identified aminoglycoside-polymyxin combinations as highly lethal. As single agents at low doses, these compounds showed little lethal action, consistent with prior reports in which lethality was seen only at high drug concentrations that are often not achieved clinically (aminoglycosides at 50–200 µg/mL for heat-shocked bacteria (68) and colistin at 10 (33) and 64 µg/mL (49) for stationary-phase cultures). Since polymyxins act by insertion into bacterial membranes (69, 70) and since aminoglycosides bind to and perturb membranes (71, 72), using a combination of the two was attractive. Members of the two compound classes were synergistic, as observed previously (33, 49, 73, 74). Although both colistin and polymyxin B, when combined with one of five different aminoglycosides, eradicated persister cells from cultures, not every antibiotic associated with membrane damage is suitable: the β-lactam ampicillin, when combined with polymyxin B, failed to kill a large proportion of persister cells. Overall, our data fit well with previous work in which a different criterion (dependence on metabolism for lethality) was used to generate synergistic combinations (33); our membrane approach explains why a polymyxin-aminoglycoside combination is exceptionally lethal.

Biofilm and phenotypically tolerant cells are also killed by an aminoglycoside-polymyxin combination, as were clinical isolates of *E. coli* and *K. pneumoniae*. With tolerant cells, the rate of killing was slow, requiring incubation for multiple days. In previous work, killing of tolerant cells occurred within a few hours, which is likely due to combinations with high doses of colistin (4 or 64 µg/mL) and amikacin/ciprofloxacin (64 µg/mL) (49). Overall, the lethal effects of the combination are quite general and may even extend to gram-positive bacteria, which are often considered to be insensitive to polymyxins (70): we observed a modest (10-fold) killing with *S. aureus* by treating with amikacin plus polymyxin. This modest effect is consistent with low, but significant, gram-positive activity of polymyxin (75).

Although it is encouraging that the combinations reduce the lethal concentration of polymyxins and aminoglycosides to where treatment is clinically feasible, enthusiasm is dampened by serious adverse effects to kidneys, especially during long-term treatment at high concentrations (69, 71, 76). We note that some renal damage, at least in animal models, arises from elevated ROS levels (77) since cotreatment with anti-oxidants decreases nephrotoxicity (69, 77, 78). Application of anti-oxidants, such as vitamin C (78), to mute nephrotoxicity should not reduce the anti-persister effect of aminoglycoside-polymyxin combinations because the lethal effect is independent of ROS. Moreover, our toxicity experiment with cultured human cells indicates that the polymyxin-aminoglycoside combination is not universally cytotoxic, as we saw no toxicity with a human cell line at doses 5- to 20-fold higher than needed for sterilization of persister *E. coli* cultures and 4- to 5-fold lower than peak serum concentrations found during clinical use (69, 79, 80).

The implications of the present work are limited in scope by the *in vitro* nature of the work, examination of only two persister mutants (*hipA7* and *metG2*), use of only one growth medium, and testing of only three gram-negative species. Moreover, we did not examine persisters present during outgrowth from stationary phase. Nor did we enrich the rare persister cells in wild-type cultures, although we did include them in survival measurements of bulk cultures. We note that a consequence of the subpopulation status of persister cells is a sharp, antibiotic-mediated drop in survival followed by a slower decline; in contrast, tolerance is characterized by a gradual decline in survival (5). Complexities are expected. For example, some *nuo* mutants exhibit survival kinetics characteristic of persisters, while others show the complete survival characteristic of tolerance (63).

We conclude by reiterating the potential importance of tolerance and persistence. Among the clinical examples are two *hipA* persister mutations (27) and a tolerance mutation in *ptsI* (81) that have been detected in *E. coli* isolates from urinary tract and blood stream infections, respectively. Moreover, a growth defect form of tolerance has been recovered from an *S. aureus* infection (19). Since laboratory evolution experiments readily generate persistent and tolerant mutants with defects in genes, such as *hipA* (25), *metG* (20, 29), *nhaA* (50), *cyaA,* and *crp* (6), that are involved in the metabolic response to lethal agents, persistence and tolerance likely emerge with high probability. Moreover, both tolerance and persistence promote the emergence of antibiotic resistance (19, 20). Particularly concerning is the finding that genetic tolerance can extend beyond antibiotics to disinfectants and antibacterial compounds used by the immune system (6). A hopeful sign is that membrane-active combinations are a promising approach for addressing problems caused by persistence and tolerance.

## MATERIALS AND METHODS

Standard microbiological methods were employed. Bacterial survival was measured by colony counting on antimicrobial-free agar plates following dilution and incubation. In many experiments, agar contained the anti-ROS agents bipyridyl and DMSO. Bacterial strains were constructed by P1-mediated transduction and CRISPR. ROS, DNA breakage, and translational activity in bulk and single cells were measured by flow cytometry and microscopy. All experiments involved at least three biological replicates; similar results were obtained in each. Statistical comparisons between two groups were assessed by using two-tailed Student’s *t*-test. For a detailed description of the methods, please see the Supplemental Material.

## SUPPLEMENTAL MATERIAL

Supplemental Material mbio.01199-25-s0001.pdf Supplemental methods, figures, and tables.

ASM does not own the copyrights to Supplemental Material that may be linked to, or accessed through, an article. The authors have granted ASM a non-exclusive, world-wide license to publish the Supplemental Material files. Please contact the corresponding author directly for reuse.
