# Supplementary material for: Antibiotic-persistent bacterial cells exhibiting low-level ROS are eradicated by ROS-independent membrane disruption
Source: mBio. 2025 Jun 30;16(8):e01199-25. doi: 10.1128/mbio.01199-25 (PMC12345145; doi:10.1128/mbio.01199-25)
Supplement: Supplemental Material — Supplemental methods, figures, and tables. [file mbio.01199-25-s0001.pdf]

Supplemental Material for

Antibiotic-persistent bacterial cells exhibiting low-level ROS are eradicated by ROS-independent membrane disruption

Yanghui Ye[a,1], Yuanqing Tian[a,1], Mingxin Duan[a,1], Weiwei Zhu[b], Jingyun Wu[a], Yilin Chen[a], Feng Xu[d], Xilin Zhao[b], Karl Drlica[c], Yuzhi Hong[a,d,*]

[a] MOE Key Laboratory of Geriatric Diseases and Immunology, Suzhou Key Laboratory of Pathogen Bioscience and Anti-infective Medicine, Institute of Molecular Enzymology, School of Life Sciences, Soochow University, 199 Ren-Ai Road, Suzhou, Jiangsu Province 215123, China

[b] State Key Laboratory of Vaccines for Infectious Diseases, Xiang-An Biomedicine Laboratory, National Innovation Platform for Industry-Education Integration in Vaccine Research, School of Public Health, Xiamen University, 4221-117 South Xiang-An Road, Xiamen, Fujian Province 361102, China.

[c] Public Health Research Institute and Department of Microbiology, Biochemistry & Molecular Genetics, New Jersey Medical School, Rutgers Biomedical and Health Sciences, Rutgers University, 225 Warren Street, Newark, NJ 07103, USA.

[d] Jiangsu Provincial Medical Innovation Center of Trauma Medicine, Key Laboratory of Alkene-carbon Fiber-based Technology & Application for Detection of Major Infectious Diseases, Institute of Trauma Medicine, Department of Emergency Medicine, the First Affiliated Hospital of Soochow University.

[1] These authors contributed equally.

Correspondence to: yzhong@suda.edu.cn (Y. Hong)

This PDF file includes:

Methods and Materials

Supplementary Figures

FIG S1. Lethality of ciprofloxacin with wild-type *E. coli*.

FIG S2. Effect of time in stationary phase on ciprofloxacin-mediated killing of *E. coli*.

FIG S3. Time-kill response of *E. coli* to various concentrations of ciprofloxacin.

FIG S4. Failure of antibiotics to kill stationary-phase cultures of *hipA7* and *metG2* mutants.

FIG S5. High-level persistence of *metG2* mutant to antimicrobials in rich medium.

FIG S6. Genetic complementation of *katGE* deficiency.

FIG S7. Ciprofloxacin-mediated death of phenotypically tolerant cells suppressed by blockage of ROS accumulation.

FIG S8. Effect of AhpCF peroxidase deficiency on killing by ciprofloxacin.

FIG S9. Nutrient deprivation and *hipA7* persistence suppress ROS accumulation during and after ciprofloxacin treatment.
FIG S10. Ciprofloxacin treatment of *E. coli* persister cells enriched by incubation with ampicillin.
FIG S11. Growth of ampicillin-enriched cells and stationary-phase cultures after dilution to nutrient-rich medium.
FIG S12. HipA7 persister cells enriched by ampicillin survive ciprofloxacin with little DNA breakage.
FIG S13. HipA7 persister cells show low-level translational activity.
FIG S14. Combinations of polymyxin with various clinically used aminoglycosides kill persister and wild-type *E. coli* cells.
FIG S15. Eradication of persister cells by combination of polymyxin B with kanamycin but not with ampicillin or ciprofloxacin.
FIG S16. Effect of antibiotic combinations on survival of wild-type and *hipA7* cultures,
FIG S17. Combination of kanamycin and polymyxin B eradicates tolerance-associated mutants.
FIG S18. Long incubation periods allow a polymyxin-amikacin combination to kill stationary-phase, tolerant cultures in the absence of dilution into fresh nutrient medium.
FIG S19. Polymyxin-amikacin combination reduces persistent survival of clinical isolates of *E. coli* and *K. pneumoniae* and a laboratory strain of *S. aureus*.
FIG S20. Killing biofilm bacteria by a combination of kanamycin and polymyxin B.
FIG S21. Bacteriostatic synergism between kanamycin and polymyxins.
FIG S22. Eradication of persister cells by a polymyxin B-kanamycin combination is independent of ROS.
FIG S23. Aminoglycoside-polymyxin combination damages membranes of persister cells.

Supplementary Tables

Table S1. Bacterial strains used in the study.
Table S2. Susceptibility (MIC) of strains to antimicrobials in the study.
Table S3. Primers used in the study.

Supplementary References

Methods and Materials

Bacterial strains and reagents

Escherichia coli K12 BW25113 strains and the clinical bacterial isolates utilized in this work are listed in Table S1. Primers used for DNA sequence amplification are presented in Table S3. Strain construction was performed by bacteriophage P1-mediated transduction (1) and CRISPR-Cas9-guided genome editing as outlined below. Luria-Bertani (LB) medium components and agar powder were procured from Sinopharm (Beijing, China). Bipyridyl and dimethyl sulfoxide (DMSO), used as antioxidants, were sourced from Aladdin Biotech (Shanghai, China). Flow cytometry reagents were obtained from Becton Dickinson Company (Franklin Lakes, NJ). Fluorescent chemical probes, including 5(6)-carboxy-2',7'-dichlorodihydrofluorescein diacetate (carboxy-H2DCFDA), 3,3′-dipropylthiadicar-bocyanine iodide (DiSC3(5)), and propidium iodide (PI), were obtained from Thermo Fisher Scientific (Waltham, MA). Antibiotics, which included ampicillin, ciprofloxacin, polymyxin B, colistin, mitomycin C, kanamycin, amikacin, streptomycin, and tobramycin, were purchased from Aladdin Biotech, Energy Chemistry, and Macklin (Shanghai, China).

Susceptibility determinations

The minimal inhibitory concentration (MIC) for each antibiotic was determined using a two-fold serial dilution method. Bacterial cultures, grown to mid-log phase in LB liquid medium, were diluted 1,000-fold to approximately 1×10^5 cells/mL. These diluted cultures were treated with each antibiotic at a variety of concentrations and incubated at 37 °C for 20–24 h. The MIC was determined as the lowest antibiotic concentration that inhibited bacterial growth by >95% relative to an untreated control, as gauged by culture turbidity. MIC determinations are listed in Table S2.

Bacteriostatic interaction

Values of the Fractional Inhibitory Concentration Index (FICI) (2) for kanamycin and polymyxin B/colistin (polymyxin E) were calculated according to FIC index = $V_{Kan}/MIC_{Kan} + V_{Pol}/MIC_{Pol}$, in which V_{Kan} and V_{Pol} are the MIC values for each antibiotic in the combination, and MIC_{Kan} and MIC_{Pol} are the MIC values of each drug administered individually.

Measurement of persistence

Overnight cultures of *E. coli* were diluted 200-fold into fresh LB broth and grown for 12–20 h to stationary phase. Cultures, typically less than 5 mL, were placed in 60-mL tubes and incubated at 37 °C under aerobic conditions with continuous shaking at 160 rpm. Cultures were diluted 20-fold into fresh LB medium supplemented with antibiotic (ciprofloxacin (20 MIC, MIC = 0.03 μg/mL); ampicillin (20 MIC, MIC = 6 μg/mL); kanamycin (2.5 MIC, MIC = 8 μg/mL; and mitomycin C (3 MIC, MIC = 1.2 μg/mL). Then samples of 0.2–1 mL were taken at various times. Cells were washed twice with 1 mL 0.9% NaCl (saline) using vortex mixing, concentration by centrifugation (9,000 g for 20 s), and resuspension in 1 mL saline to remove residual antibiotic. Serial dilution was performed in 0.9% NaCl, and the resulting preparations were then spotted on LB agar or on agar containing 0.3 mM bipyridyl and 5% DMSO. Colony-forming units (CFUs) were determined after incubation at 37 °C for 3 days. Cells failing to form colonies were considered to be dead. Percent survival was determined relative to samples taken at the time of antibiotic addition.

Treatment with two antibiotics

Overnight cultures of *E. coli* wild-type, *hipA7,* and *metG2* cells were diluted 200-fold into fresh LB broth and grown for 12–20 h to stationary phase. Cultures were diluted 20-fold into fresh LB medium containing combinations of two antibiotics (20 MIC ciprofloxacin plus 20 MIC ampicillin; 20 MIC ciprofloxacin plus 3 MIC kanamycin; 20 MIC ciprofloxacin plus 3 MIC mitomycin C; 20 MIC ampicillin plus 3 MIC kanamycin; 20 MIC ampicillin plus 3 MIC mitomycin C; 3 MIC kanamycin plus 3 MIC mitomycin C). Single antibiotic treatment was included for comparison. Then 0.2 to 1 mL aliquots were taken at various times, concentrated by centrifugation (9,000 g for 40 s), and washed twice with 1 mL saline using vortex mixing to remove residual antibiotic. Cells were resuspended in saline at a volume equal to that of the prewashed cultures. Serial dilution was performed in saline, and the resulting diluted preparations were spotted on LB agar. Survival was determined at multiple times by visual colony counting after incubation at 37 °C for 3 days.

Variation of cell density

Cell density was varied in two ways. In one, stationary-phase cells were diluted into various volumes of fresh medium and then treated with ciprofloxacin at 20 MIC for 10 h. In a second method, log-phase cells were prepared by a 200-fold dilution into fresh LB broth and grown to OD_{600} = 0.3. Cells were collected by centrifugation at 9,000 g for 20 s and then suspended in various volumes of LB broth. These cultures were then treated with 20 MIC ciprofloxacin.

Nutrient deprivation by resuspension in saline

Stationary-phase, wild-type cultures were diluted 200-fold into fresh LB both and grown to early log phase (OD_{600} = 0.3). Cells were washed twice in 1 mL 0.9% saline using centrifugation and resuspension to remove external nutrients. The washed cells were resuspended in the pre-wash volume of saline for nutrient deprivation and incubated at 37 °C. After undergoing nutrient deprivation for either 24 or 48 h, cells were treated with ciprofloxacin at a concentration of 20 MIC and incubated for another 5 h. Simultaneously, aliquots of the nutrient-deprived cells (after starvation for 24 or 48 h) were collected via centrifugation, and they were re-incubated in fresh LB medium containing 20 MIC ciprofloxacin for an additional 5 h.

Killing of *E. coli* and *K. pneumoniae* biofilm cells

Biofilms composed of *E. coli* (strain 0001) or *K. pneumoniae* (strain 0066) were prepared by allowing cultured cells to adhere to walls of a plastic culture dish. Overnight cultures, grown in LB medium, were diluted 1:10,000 to ~1×10^5 CFU/mL in fresh LB medium. Aliquots of 1 mL were distributed to wells of a 24-well plastic culture plate (Shuohua Company, China) and incubated at 37 °C for 40 h. Liquid medium and unattached cells were removed, and each well was washed three times with 0.9% NaCl. The cells in biofilm were then treated with 2.5 MIC kanamycin plus 3 MIC polymyxin B in fresh LB broth for 25 h. The wells at various treatment times were sonicated with a digital-controlled ultrasonic processor (ShuMei Company, China) at 100 W for 10 min to detach cells from the well wall. The detached cells were washed by centrifugation-resuspension, diluted serially, and plated on drug-free agar to determine CFU.

CRISPR-induced genome editing

Gene deletions and single-site edits in *E. coli* DNA using the CRISPR-Cas9 system were as described (3, 4). Briefly, the temperature-sensitive pCas vector carrying the *cas9* gene was introduced into target strains via electroporation. Strains harboring the pCas vector were cultivated at 30 °C and prepared as competent cells by washing three times using ice-cold 10% glycerol. Subsequently, the pTargetF vectors, which hosted each specific single-guide RNA (sgRNAs) and/or a DNA fragment designated for homologous recombination, were transformed into the pCas-harboring cells by electroporation. The N20 sequences and primers used for cloning these DNA segments are listed in Supplementary Table 3. Following completion of genome editing, the pCas and pTargetF plasmids were eliminated from the *E. coli* cells as described (3). The accuracy of the target sequences within the genetically modified cells was verified by polymerase chain reaction (PCR) amplification followed by sequencing performed by Genewiz (Suzhou, China).

Complementation of *katGE* deficiency

A *katE* fragment containing its native promoter and a Cm^R-p15Aori fragment in the low-copy vector pACYC184 were amplified by PCR using primers listed in Table S3. The two fragments were ligated following digestion with *Xho*I and *Spe*I, generating the recombinant plasmid pACYC-*katE*. The *katE* fragment was sequenced to verify its presence. pACYC-*katE* was introduced by electroporation (Bio-Rad, USA) into Δ*katGE* and *hipA7* Δ*katGE* mutants for complementation of catalase deficiency. The plasmid in cells was maintained by the presence of 20 μg/mL chloramphenicol. However, chloramphenicol was absent during growth following 1:200 dilution to stationary phase and during antibiotic killing for determination of persistence.

Enrichment of *hipA7* persister cells

Ampicillin was used to lyse non-persister cells in a culture under nutrient-rich conditions. For *hipA7* persisters, stationary-phase cultures were obtained by growth for 12–20 h and then diluted 20-fold into fresh LB broth supplemented with 20 MIC ampicillin. These ampicillin-containing cultures were incubated at 37 °C with shaking for 10 h, after which persister cells were collected by centrifugation at 9,000 g for 1 min. They were washed twice with 0.9% saline and resuspended in fresh LB medium at the pre-wash volume. They were then treated with 10 MIC ciprofloxacin followed by incubation at 37 °C for 10 h. When appropriate, fluorescent dyes were added at the start of ciprofloxacin treatment to monitor molecular events. For example, carboxy-H2DCFDA was used to indicate accumulation of reactive oxygen species (ROS) (5-7), DiSC3(5) demonstrated membrane depolarization, and propidium iodide identified membrane rupture and cell death. We note that persister enrichment for microscopy was not applied to the wild-type strain because extensive killing with the strain (10^5-fold loss of survival) by ampicillin generates a large number of cell fragments that confound visual imaging of a few persister cells.

Measurement of ROS

To assess intracellular ROS levels, cells were stained using the fluorescent ROS indicator carboxy-H2DCFDA (8). These cells were in stationary-phase cultures, cultures diluted into fresh LB broth, as well as the *hipA7* persister cells enriched using ampicillin. Carboxy-H2DCFDA was added to cultures at a final concentration of 10 μM. Cells subjected to a variety of treatments were collected by centrifugation at 9,000 g for 1 min, washed twice with 1 mL 0.9% saline, resuspended in saline, and then chilled on ice. Fluorescence intensities, which reflect intracellular ROS levels, were measured using flow cytometry and fluorescence microscopy.

To assess ROS level present after antibiotic removal, cells treated with antibiotic were washed twice using saline and centrifugation. Cells were then resuspended in fresh, antibiotic-free LB medium containing 10 µM carboxy-H2DCFDA, followed by incubation at 37 °C for 3 h. Fluorescence quantification was performed on aliquots of these cultures.

Visualization of DNA Breakage

Intracellular double-stranded DNA breakage in cultured cells was detected using RecN-YFP (6, 9). The gene construct *recN-yfp::tet* (10) was integrated into both the *hipA7* mutant and wild-type strains in place of native chromosomal *recN* via P1 phage-mediated transduction. These *recN-yfp*-encoding strains were treated with ciprofloxacin. Following treatment, cells were rinsed with saline and placed on an agarose pad (11) for visualization. The formation of green fluorescent foci, indicative of DNA breakage, was observed using fluorescence microscopy.

Measurement of translation in persister and phenotypically tolerant cells

Global translation activity was assessed by the expression of *rplA*, which encodes the L1 ribosomal protein. An *rplA-yfp::cat* DNA fragment was introduced from an *E. coli* strain (0182) into the *hipA7* mutant using P1 phage-mediated transduction to replace the native chromosomal *rplA* gene. The intensity of RplA-YFP fluorescence was subsequently quantified using flow cytometry and fluorescence microscopy.

Evaluation of membrane damage

The fluorescent dyes DISC3(5) (12) and propidium iodide (13) were used to evaluate reduction of membrane potential and membrane damage, respectively. The assays included the addition of either 2.5 µM final concentration of DISC3(5) or 5 µM final concentration of propidium iodide into cultures 10 min before treatment with antibiotic. Fluorescence intensities were then measured by flow cytometry and fluorescence microscopy.

Flow cytometry

Antibiotic-treated cells were collected by centrifugation at 9,000 g for 1 min, followed by two washes, each using 1 mL of 0.9 % saline. Cells were then resuspended in saline at 1–5 × 10^6 cells/mL. Sample tubes were wrapped in aluminum foil to prevent light exposure and stored on ice. Bacterial fluorescence intensity was quantified using flow cytometry. Various indicators were present in the cultures for the detection of specific features: carboxy-H2DCFDA (10 µM) for determining intracellular ROS, DiSC3(5) (2.5 µM) for measuring membrane potential, and PI (5 µM) for identifying membrane damage. To assess auto-fluorescence, a sample lacking dye was also included. A total of 200,000 ungated events for each sample was determined using a BD Accuri C6 Plus flow cytometer (Becton Dickinson, USA). Detection parameters included 20 mV laser power, 533/30 nm band-pass filter (FL1-channel) for observing carboxy-H2DCFDA and YFP, a 585/40 nm band-pass filter (FL2-channel) for PI, and a 670 LP filter (FL3-channel) for DiSC3(5). The acquired data were then evaluated using BD Accuri C6 software.

Fluorescence microscopy

Microscopy was performed using a Nikon Ti2-E inverted microscope (Nikon, Japan) that was equipped with differential interference contrast (DIC) for viewing unstained structures of cells and with filters for the excitation and emission of YFP, carboxy-H2DCFDA, DiSC3(5), and propidium iodide. Additional features included a motorized stage and integration of the pco.edge 4.2 bi sCMOS camera (Kelheim, Germany). Concentrated cell

samples were spotted onto an agarose pad on a microscope slide (11). Automated unbiased image acquisition was carried out utilizing the Nikon NIS Elements BR imaging software.

Potential antibiotic toxicity for mammalian cells

We utilized the Cell Counting Kit-8 (CCK-8) assay (Topscience Biotech, China) to assess potential cytotoxic effects of antibiotics on human cells following the manufacturer's protocols. The THP-1 cell line, a human monocytic leukemia lineage, was propagated in RPMI 1640 medium (BasalMedia, Shanghai) supplemented with 10% fetal bovine serum (Cell-Box, Changsha, China). Cultured cells were seeded at a density of 5×10^5 cells/mL into 96-well plates and incubated at 37 °C for 12 h. Then cells were subjected to one of several treatment regimens for a 24-h period: kanamycin at 80 μg/mL, amikacin at 80 μg/ml, polymyxin B at 6 μg/mL, carbonyl cyanide m-chlorophenylhydrazone (CCCP) at 20 μg/mL, a combination of kanamycin (80 μg/mL) with polymyxin B (6 μg/mL), and finally a mix of amikacin (80 μg/mL) with polymyxin B (6 μg/mL). Subsequently, 10 μl of the CCK-8 solution from the assay kit was added to each well, followed by an additional hour of incubation. Absorbance readings were then determined at 450 nm using a microplate reader with minimal light exposure.

Statistical considerations

At least three biological replicates were obtained for all experiments. Each data point represents the mean of replicate experiments; error bars show standard deviation, unless otherwise stated. Statistical comparisons between two groups were assessed by using two-tailed Student's *t*-test. $p \leqslant 0.05$ and lower were considered significant. *p*-values were usually not presented for cell survival with kinetic trends having greater than 10-fold differences.

Data and materials availability

Strains and data supporting the findings of this study are available from the corresponding author upon reasonable request.

SUPPLEMENTARY FIGURES

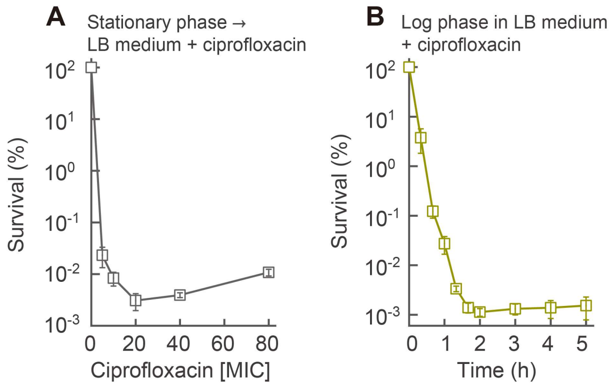

FIG S1. Lethality of ciprofloxacin with wild-type *E. coli*.

When stationary-phase cultures of wild-type *E. coli* were treated with various concentrations of ciprofloxacin for 4 h following a 20-fold dilution into fresh rich medium, survival dropped to 0.005–0.02% (Panel ***A***). An optimal bactericidal concentration, which is characteristic of all quinolones, is seen at 20 MIC for ciprofloxacin. The loss of killing at high concentration is attributed to the Eagle effect, which depends on the Lon protease (14) and is associated with a loss of ROS (15, 16). The Eagle effect is less pronounced with fluoroquinolones than with first-generation quinolones (14), probably because the former have an additional ROS-independent mode of killing (16). That mode of killing, which is not blocked by chloramphenicol, an inhibitor of ROS production (8), likely involves chromosome fragmentation (17, 18). The contribution of this mode to killing is likely suppressed by DNA repair; plating on anti-oxidant-containing agar is needed to determine its contribution relative to the ROS-dependent mode (19).

When a log-phase culture (OD_{600} = 0.3) was incubated with ciprofloxacin, persistent survival was 0.001% (panel ***B***), a value similar to that seen stationary-phase cultures diluted into ciprofloxacin-containing LB medium (Fig. 1A in main text).

Details: (*A*) Effect of ciprofloxacin concentration on survival. Cultures of wild-type *E. coli* strain (0001, *n*=3) grown for 20–24 h, were diluted 20-fold into fresh LB medium containing 5, 10, 20, 40 or 80 MIC ciprofloxacin. After treatment for 4 h, cells were washed twice by centrifugation using 0.9% NaCl, and they were then plated on standard LB agar for CFU determination. (*B*) Survival of log-phase cultures during ciprofloxacin treatment. Stationary-phase wild-type cultures (*n*=3) were diluted 200-fold into fresh LB broth and grown to a cell density of OD_{600} = 0.3. The cells were then treated with 20 MIC ciprofloxacin for the indicated times, and CFU was accessed as in panel *A*. *n* denotes the number of independent biological replicates. Data represent the mean ± SD.

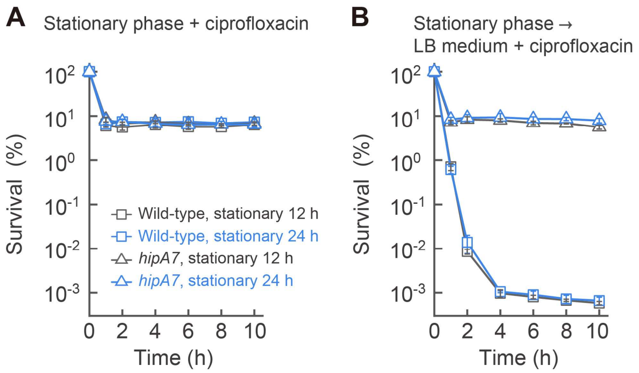

FIG S2. Effect of time in stationary phase on ciprofloxacin-mediated killing of *E. coli*.

When *E. coli* cultures (wild type or *hipA7* mutant) were kept in stationary phase for the indicated times (12 or 24 h) and treated with 20 MIC ciprofloxacin, time in stationary phase was found to have no effect on survival (***A***). Dilution of stationary-phase cells into rich medium containing 20 MIC ciprofloxacin led to a drop in survival for wild-type cells (***B***). Survival of the *hipA7* mutant (***B***) was unaffected by restoration of growth or by the time in stationary phase.

Details. Stationary-phase cultures of wild-type (strain 0001, *n*=3) or *hipA7* (strain 0022, *n*=3) cells were prepared by growth for 12 h or 24 h before treatment with 20 MIC ciprofloxacin for the indicated times (*A*) or after a 20-fold dilution into fresh LB medium containing 20 MIC ciprofloxacin for the indicated times (*B*). Samples were taken, washed by centrifugation-resuspension, and plated on LB agar for CFU measurement. Data represent the mean ± SD.

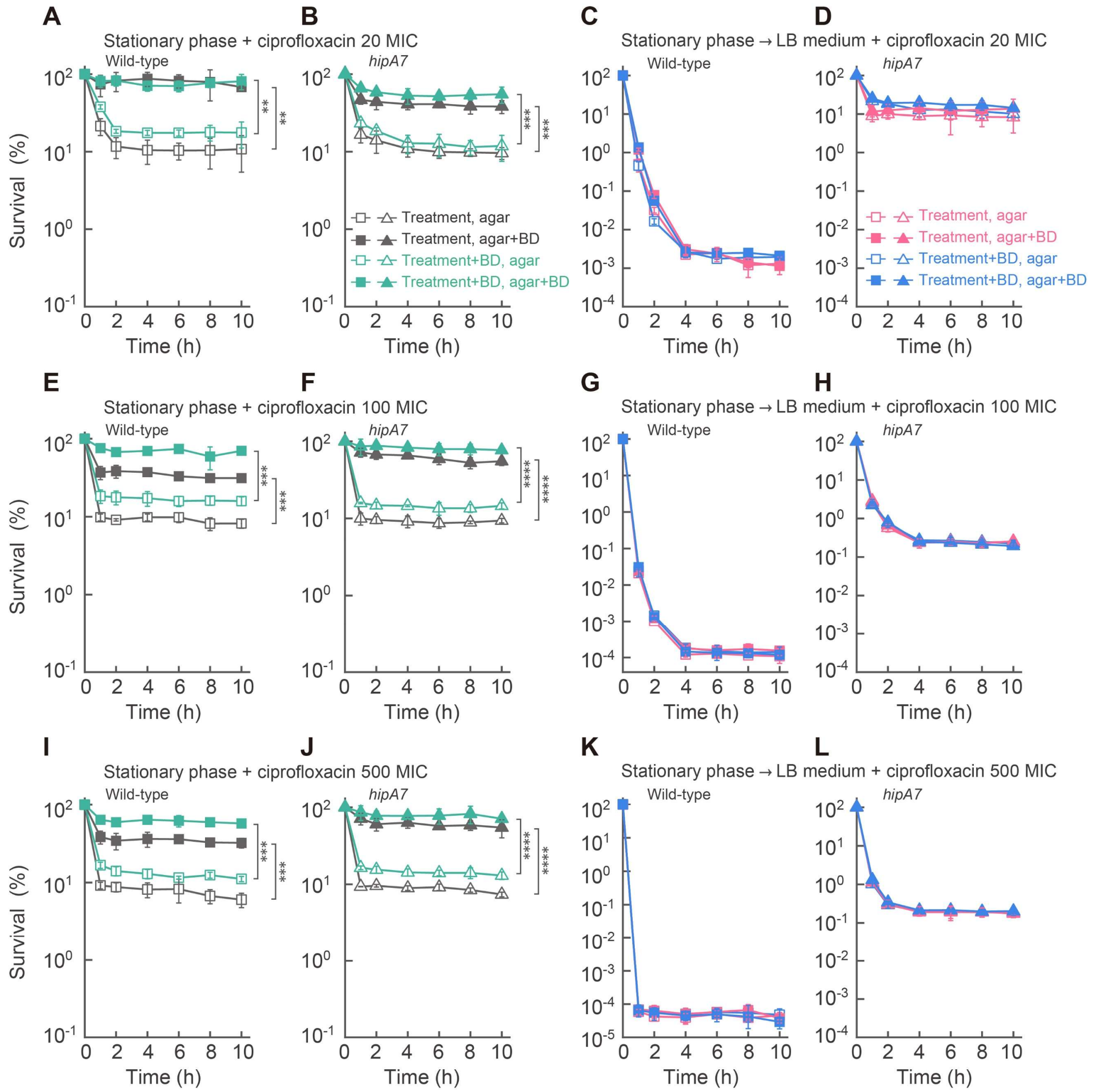

FIG S3. Time-kill response of *E. coli* to various concentrations of ciprofloxacin.

Several concentrations of ciprofloxacin (20, 100, and 500 MIC) were examined for concentration-dependent effects on survival during stationary phase and after release from stationary phase. When wild-type, stationary-phase cultures were treated with ciprofloxacin (***A, E, I***), anti-oxidants in the cultures increased survival slightly, and anti-oxidants in both cultures and on agar survival plates brought survival to 100%. The effect of antioxidants is interpreted as ROS-mediated killing in the presence of ciprofloxacin or after its removal (7, 19). The antioxidants were less effective at the high concentrations of ciprofloxacin, probably because those concentrations cause more ROS-independent killing (see Fig. S1 legend). Survival of only 10% (***A***) rather than the 100% seen with oxolinic acid (Fig. 1B in the main text) is likely due ciprofloxacin-mediated DNA damage during stationary phase being carried over to conditions in which nutrients are restored on agar plates used to determine survival (20). First-generation quinolones do not generate detectable ROS-independent damage with wild-type cells (16-18). When ciprofloxacin was added at culture dilution, killing was rapid, and anti-oxidants had little effect (***C, G, K***). Saturation with respect to killing occurred at 100 MIC.

When the *hipA7* mutant was treated with ciprofloxacin in stationary phase (***B***), survival was 10% and an ROS effect was observed. The persister mutation was unable to completely suppress the ROS effects on survival, a result that is consistent with the persister mutation being unable to block the ROS-independent mode of damage that elicits ROS when nutrients are restored during plating on agar. It did, however, eliminate the ciprofloxacin concentration dependence (***B, F, J***). After dilution of stationary culture into rich medium (***D, H, L***), the persister mutant lost some of its ability to suppress killing at the higher drug concentrations (***H, L***; 50-fold decrease in survival). Even so, survival was still about 5,000 times higher than seen with wild-type cells. Anti-oxidants had no effect, consistent with saturating killing occurring rapidly. The loss of *hipA7*-mediated protection (***H, L***) is explained by the insensitivity of the ROS-independent mode of killing to the effects of the mutation. One implication is that persistence studies using 500 MIC ciprofloxacin may need to be re-examined due to complexity introduced by the Eagle effect and the ROS-independent mode of killing by ciprofloxacin.

Details. (*A−L*) Wild-type (0001, *n*=3) and *hipA7* (0022, *n*=3) cultures were grown to stationary phase in LB medium (20 h overnight). Cultures were diluted 20-fold into fresh LB medium or maintained as stationary-phase cultures. Ciprofloxacin at 20, 100, or 500 MIC (MIC = 0.03 µg/mL) was added to each culture, and incubation was continued for 10 h. Samples (300 µl) were taken at the indicated times, washed twice with saline using centrifugation, and plated following serial dilution on either LB agar or on LB agar plus 0.3 mM bipyridyl and 5% DMSO (BD), as indicated in the figure panels, for at least two days. Data represent the mean ± SD. **, $p<0.01$; ***, $p<0.001$; ****, $p<0.0001$.

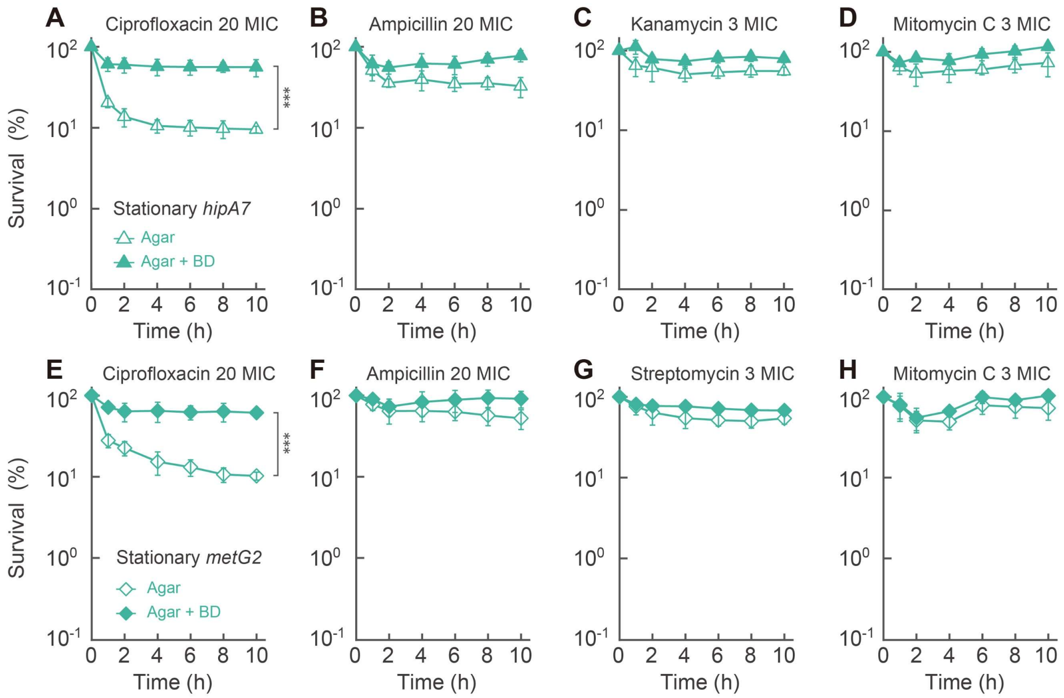

FIG S4. Failure of antibiotics to kill stationary-phase cultures of *hipA7* and *metG2* mutants.

When stationary-phase cultures of the *hipA7* mutant were treated with ciprofloxacin, survival declined to ~10%, as measured using standard LB agar. Introduction of anti-oxidants (bipyridyl and DMSO) into the agar restored full viability. No killing was observed with cells treated with members of three other antimicrobial classes (ampicillin, kanamycin, and mitomycin C) in either the presence or absence of anti-oxidants in the survival assay agar (***A*, *B***). With the *metG2* mutant, stationary-phase cultures also exhibited little death when treated with any of the four antimicrobials (***C*, *D***). These data reinforce the distinct behavior of ciprofloxacin discussed in Fig. S1 and S3 legends. They also suggest a general behavior of persister mutations that induce the stringent response.

Details. (*A, B*) Little death of stationary-phase *hipA7* cultures following treatment with a representative from each of four antimicrobial classes. Stationary-phase cultures of *hipA7* (strain 0022) were diluted 20-fold into fresh LB medium containing one of the following antimicrobials: 20 MIC ciprofloxacin, 20 MIC ampicillin, 3 MIC kanamycin, or 3 MIC mitomycin C. Survival was determined at the indicated times using both LB agar and agar containing anti-oxidants (0.3 mM bipyridyl and 5% DMSO, BD). (*C, D*) Stationary-phase *metG2* cultures maintain viability after antimicrobial treatment. The experimental conditions were as described in panels *A* and *B*, except the *metG2* strain (0024) substituted for the *hipA7* strain. *n*, which indicates number of independent biological replicates, equaled three in all cases. Data represent the mean ± SD. ***, $p<0.001$.

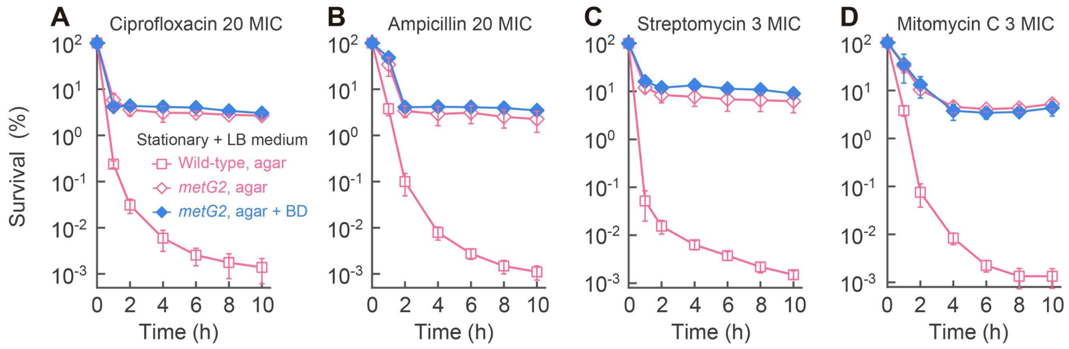

FIG S5. High-level persistence of *metG2* mutant to antimicrobials in rich medium.

When stationary-phase *metG2* cultures were shifted to nutrient-rich medium rather than remaining in stationary phase and treated with a member of four classes of antimicrobial, survival remained at 5–10%. This was 5,000–10,000 fold higher than the persistence level of the wild-type strain. Survival during treatment in rich medium was insensitive to the presence of anti-oxidants in the assay agar. This failure of anti-oxidants to increase survival is explained by rapid, extensive killing being complete before plating, as the antioxidants were present only in the assay agar.

Details. (*A−D*) Stationary-phase *metG2* cultures (strain 0024) were diluted 20-fold into fresh LB medium containing 20 MIC ciprofloxacin, 20 MIC ampicillin, 3 MIC streptomycin, or 3 MIC mitomycin C. In each panel, the wild-type strain (0001) served as a control for low-level persistence. The antioxidants were bipyridyl and DMSO. The results were from 3 independent biological replicates that had similar results. Data represent the mean ± SD.

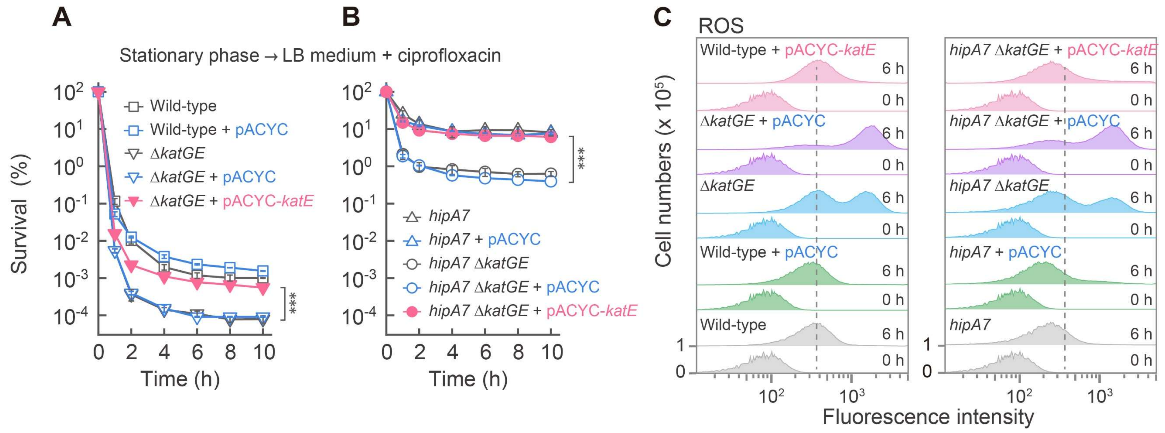

FIG S6. Genetic complementation of *katGE* deficiency.

Deletion of *katGE* lowered survival of cells grown in rich nutrients when stationary-phase cultures were diluted 20-fold into fresh LB medium and treated with ciprofloxacin for both wild-type and *hipA7* mutant cells (***A, B***). Complementation with wild-type *katE* carried by the low-copy vector pACYC184 restored survival to the wild-type level, while the empty vector showed little effect. The same phenomenon is shown in panel ***C*** where ROS was monitored by the fluorescent dye carboxy-H2DCFDA. In this assay, higher levels of ROS are indicated by shifting of the peaks to the right.

Details. (*A, B*) Stationary-phase *E. coli* cultures were treated with 20 MIC ciprofloxacin in fresh, rich medium for the indicated times and then assayed for survival by dilution and plating on agar. Data represent the mean of three independent determinations. Strains were as follows: wild-type (strain 0001), wild-type plus empty vector pACYC184 (0699), Δ*katGE* (0042), Δ*katGE* + empty pACYC184 (0702), Δ*katGE* + pACYC-*katE* (0701), *hipA7* (0022), *hipA7* + pACYC184 (0705), *hipA7* Δ*katGE* (0354), *hipA7* Δ*katGE* + pACYC (0708), and *hipA7* Δ*katGE* + pACYC-*katE* (0707). (*C*) Cultures of the indicated strains were treated with 20 MIC ciprofloxacin and carboxy-H2DCFDA for zero or 6 h (as in *A* and *B*) followed by flow cytometry. The data were representative from three independent replicates showing similar results. ***, $p<0.001$.

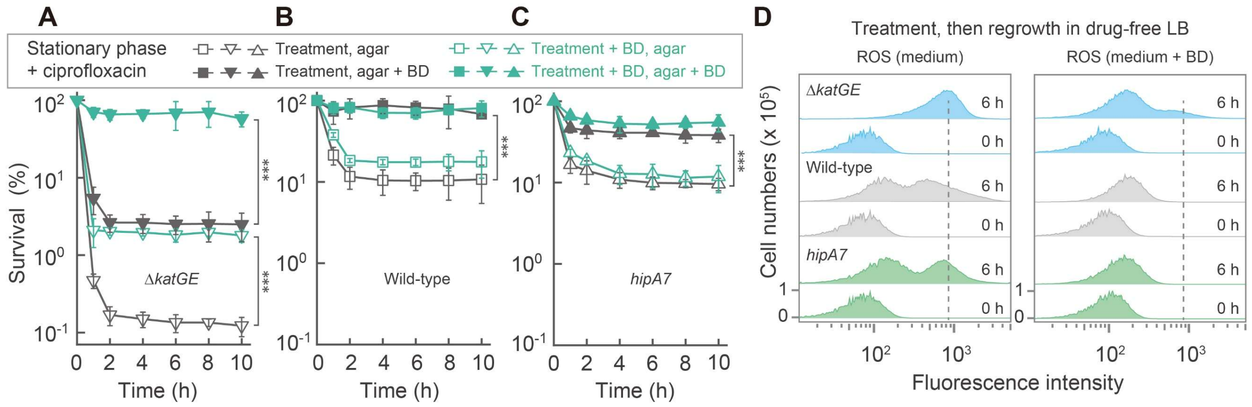

FIG S7. Ciprofloxacin-mediated death of phenotypically tolerant cells suppressed by blockage of ROS accumulation.

The elevated killing of a *katGE*-deficient mutant by ciprofloxacin during stationary phase was completely reversed by including bipyridyl and DMSO in the liquid culture medium and the assay agar (***A***). With strains containing wild-type *katGE*, anti-oxidants present only in agar but absent during treatment were able to suppress death (***B***, ***C***). ROS signal was largely suppressed by anti-oxidants during regrowth of the ciprofloxacin-treated cells in drug-free medium (***D***). The results support the conclusion that the catalase deficiency increases killing by allowing ROS to accumulate during ciprofloxacin treatment and after removal of the antibiotic.

Details. (*A−C*) Antioxidants suppressed catalase-deficiency-elevated killing of stationary-phase cultures. Stationary-phase cultures of the WT (strain 0001), Δ*katGE* (0042) and *hipA7* (0022) were treated with 20 MIC ciprofloxacin in the presence or absence of 0.3 mM bipyridyl plus 5% DMSO (BD) as indicated in the panels. Survival was determined by plating on LB agar and agar containing bipyridyl plus DMSO as indicated in the panels. Data are presented as means ± SD (n=3). (*D*) Fluorescence indicating intracellular ROS of stationary-phase cells after ciprofloxacin removal in the presence or absence of anti-oxidants. As in *A−C*, stationary-phase cultures (n=3) were treated with 20 MIC ciprofloxacin for the indicated times, washed, and then incubated in antibiotic-free, fresh medium (regrowth) containing an ROS indicator (carboxy-H2DCFDA) for 3 h in the presence or absence of bipyridyl plus DMSO. Fluorescence was measured by flow cytometry. Representative data are shown that were taken from three independent replicates giving similar results. ***, p<0.001.

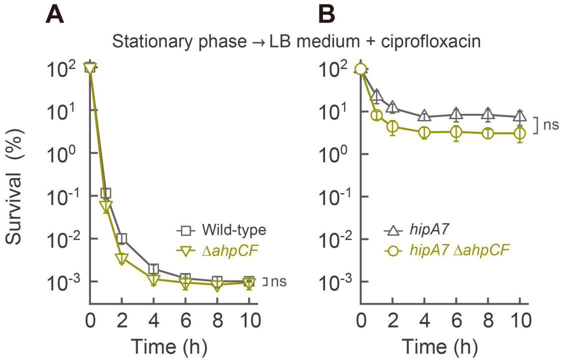

FIG S8. Effect of AhpCF peroxidase deficiency on killing by ciprofloxacin.

Deletion of the AhpCF peroxidase had little effect on the lethal activity of ciprofloxacin with *E. coli*. The data indicate that the KatG and KatE catalases are the dominant factors removing hydrogen peroxide from ciprofloxacin-treated cells.

Details. Stationary-phase *E. coli* cells were diluted 20-fold into fresh LB medium and treated with 20 MIC ciprofloxacin for the indicated times after which aliquots were plated for determination of CFU. Strains were wild-type (0001), Δ*ahpCF* (0696), *hipA7* (0022) and *hipA7* Δ*ahpCF* (0697). Data represent as means ± SD (*n*=3). ns, no significance.

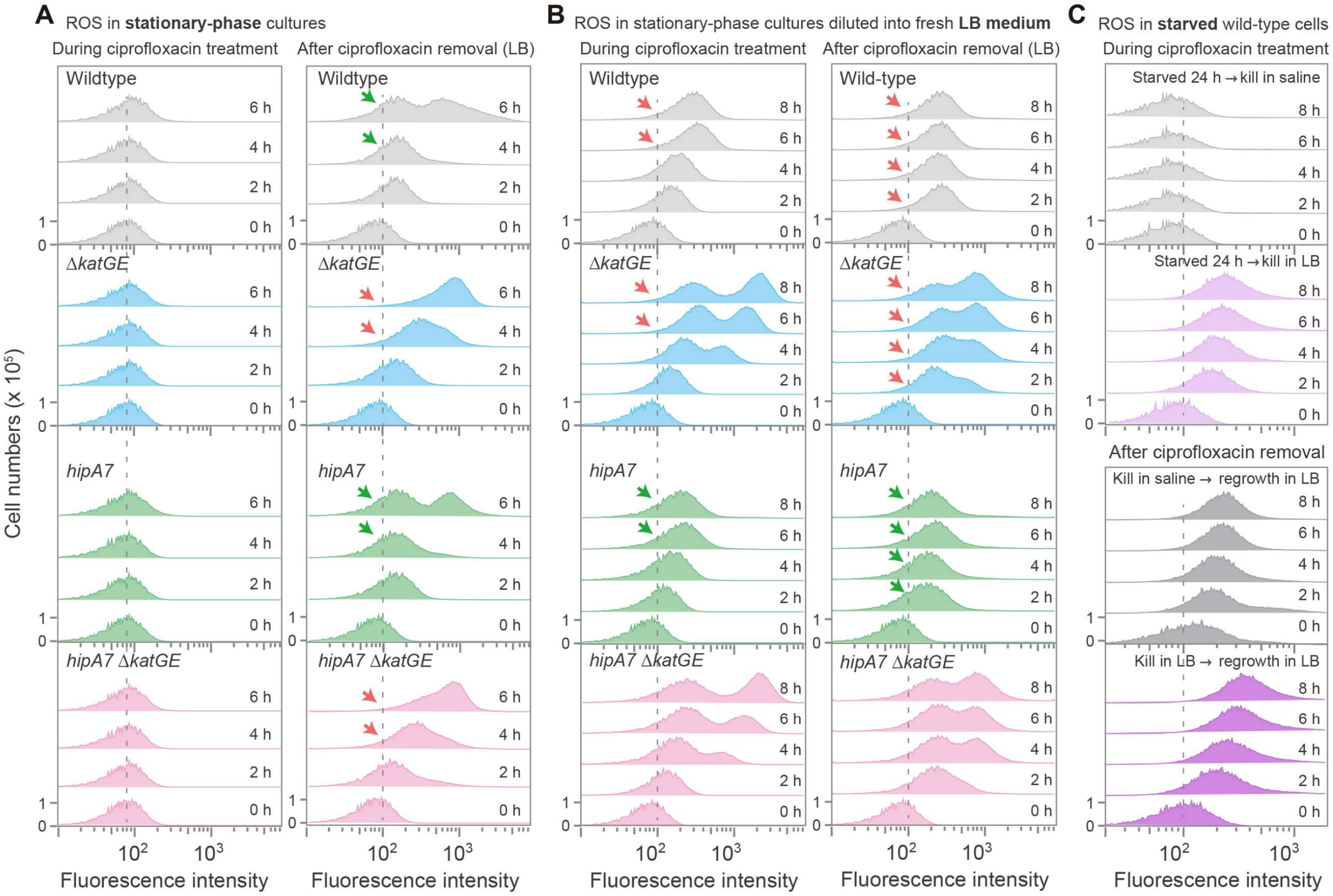

FIG S9. Nutrient deprivation and *hipA7* persistence suppress ROS accumulation during and after ciprofloxacin treatment.

Little accumulation of ROS was observed in stationary-phase cultures of the wild-type, Δ*katGE*, *hipA7,* and *hipA7* Δ*katGE* strains that were treated with 20 MIC ciprofloxacin (left portion of Panel ***A***; Fig. 3C in the main text). ROS signals were seen when these treated cells were incubated in fresh, antibiotic-free medium following removal of ciprofloxacin (right portion of Panel ***A***). A small fraction of wild-type and *hipA7* cells showed similar low ROS levels (as indicated with arrows), supporting their 10% survival with stationary-phase cultures on standard LB agar. The results indicate suppression of ROS in stationary-phase cultures and generation of ROS via ciprofloxacin-mediated DNA damage once nutrients become available.

When stationary-phase cultures of the four strains were diluted 20-fold into fresh medium containing ciprofloxacin, an increase in ROS corresponded with increased duration of treatment (left portion of Panel ***B***). A deficiency of the ROS-detoxifying catalase genes in either the *hipA7* mutant or in an otherwise wild-type strain raised ROS levels (peak shift to the right). After antibiotic removal, ROS accumulation persisted, with Δ*katGE* strains exhibiting higher levels of ROS, seen as an additional high-level peak to the right. Overall, these results fit with survival levels (Figs. 2A, 3A and 3B in the main text). Notably, a subpopulation within *hipA7* cultures displayed a lower ROS signal than the wild-type strain (arrows in Panel ***B***).

Panel ***C***. Starvation in saline resulted in a 10^4-fold increase in survival during ciprofloxacin treatment, compared to survival when equivalent cultures were subjected to treatment in a nutrient-rich medium (Fig. 1G in the main text). No accumulation of ROS was detected during addition of ciprofloxacin to wild-type cells that had been initially starved in saline for 24 h (top panel in ***C***). However, survival declined to 10% as assayed on standard agar (Fig. 1G in the main text), suggesting ROS accumulation during recovery growth on agar. Indeed, ROS signals appeared when the starved, treated cells were incubated in fresh, antibiotic-free recovery medium following removal of ciprofloxacin (the third panel in ***C***). In contrast, a pronounced ROS build-up was observed when cells, starved for 24 h, were incubated in fresh, rich medium containing ciprofloxacin (the second panel in ***C***). These results substantiate the role of nutrient deprivation in mitigating ROS accumulation and subsequent cell death. Overall, data in the figure indicate that both nutrient-deprived conditions and persistence mechanisms effectively impede toxic ROS accumulation.

Details. (*A*) Suppression of ROS by stationary phase was released by fresh medium. Stationary-phase cultures of the wild-type (strain 0001), Δ*katGE* (0042), *hipA7* (0022), and *hipA7* Δ*katGE* (0354) strains (*n*=3, each) were treated with 20 MIC ciprofloxacin plus 10 μM carboxy-H2DCFDA. ROS was assessed at the specified times. Parallel cultures, treated with ciprofloxacin in the absence of carboxy-H2DCFDA, were washed and incubated in fresh medium containing carboxy-H2DCFDA but lacking ciprofloxacin for 3 h for flow cytometry examination. (*B*) Increase in ROS levels due to catalase deficiency during and after ciprofloxacin removal. Stationary-phase cultures of the four strains (*n*=3, each) were treated as in panel *A* but in the presence of 20-fold volumes of fresh LB medium. Left and right panels show ROS signals during and after treatment, respectively. (*C*) ROS suppression during nutrient starvation and accumulation after ciprofloxacin removal. Log-phase (OD_{600} = 0.3) cultures of the wild-type strain (0001; *n*=3), following two rounds of washing by centrifugation, were incubated in saline (0.9% NaCl) for 24 h. Subsequently, ciprofloxacin and carboxy-H2DCFDA were added. Aliquots of the 24-h-starved cells were also collected and incubated in LB medium containing ciprofloxacin plus carboxy-H2DCFDA. ROS levels were analyzed using flow cytometry following washing by centrifugation and resuspension. In parallel, the 24-h starved cells (*n*=3) were treated with ciprofloxacin in saline or fresh LB medium lacking carboxy-H2DCFDA for the indicated times. Upon washing, the treated cells were incubated in fresh LB medium containing only carboxy-H2DCFDA for 3 h for ROS analysis. *n* is the number of independent biological replicates; one representative result is shown.

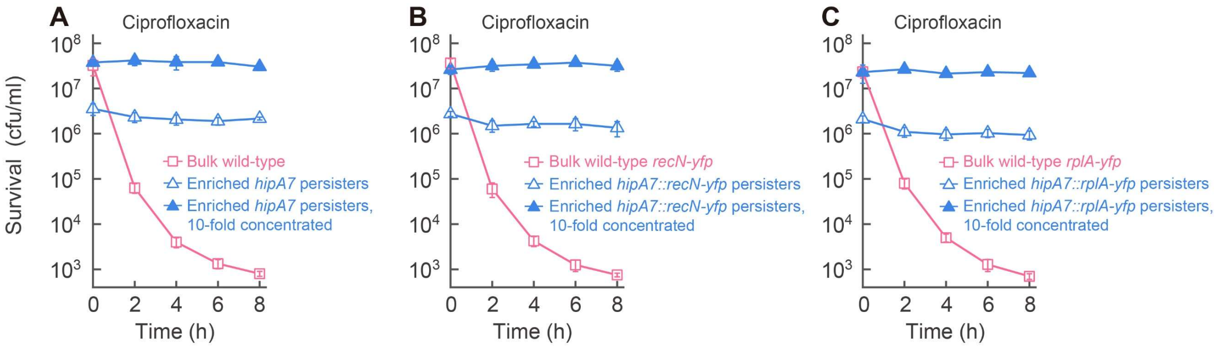

FIG S10. Ciprofloxacin treatment of *E. coli* persister cells enriched by incubation with ampicillin.

E. coli persister cells were enriched by elimination of the dominant, non-persister population by ampicillin treatment. When enriched persisters were concentrated 10-fold, the concentration of the cells equaled that of the culture prior to enrichment. Since the persister subpopulation is about 10% of the total, these data indicate that all persister cells are viable (data at t=0 indicates that the yield of enriched persister cells was approximately100%, Panels ***A*−*C***). The inability of ciprofloxacin to kill enriched cells demonstrates tolerance to the drug. A chromosomal fusion of *yfp* to native *recN* and *rplA* had little effect on cell persistence (***B*** and ***C***).

<u>Details</u>. *E. coli* cultures were grown overnight to stationary phase and 1) diluted 20-fold into fresh LB medium containing 20 MIC ciprofloxacin, or 2) diluted 20-fold and enriched by a 10-h treatment with 20 MIC ampicillin before treatment with ciprofloxacin, or 3) enriched and then concentrated 10-fold before treatment with ciprofloxacin. Strains shown in the panels were (*A*) *hipA7* mutant (0022, *n*=5), (*B*) *hipA7::recN-yfp* (0140, *n*=5), (*C*) *hipA7::rplA-yfp* (0355, *n*=3). At the indicated times, samples were removed for determination of CFU by plating and colony counting. Data represent means ± SD.

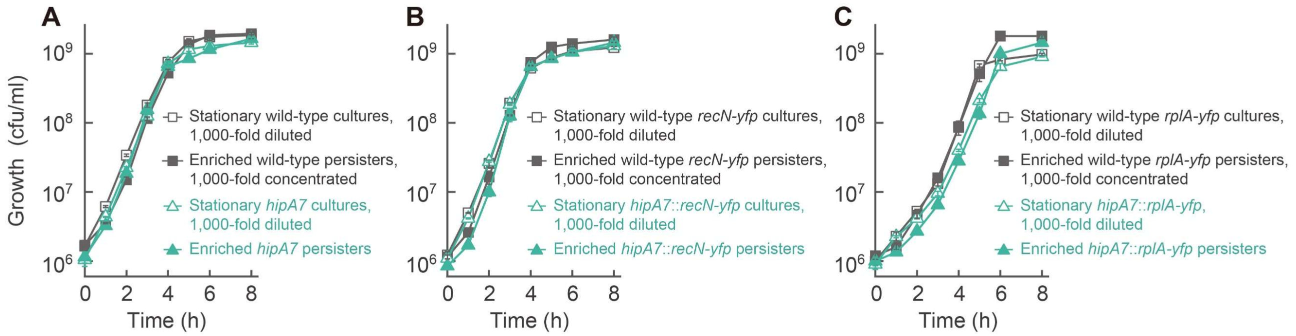

FIG S11. Growth of ampicillin-enriched cells and stationary-phase cultures after dilution to fresh medium.

Wild-type and *hipA7* mutant persister strains exhibited little lag upon dilution of stationary-phase cultures into fresh medium, and little difference was seen between the two strains (Panels ***A−C***). After enrichment of persisters by ampicillin treatment, *hipA7* persisters behaved like their bulk populations and like wild-type populations. No difference was seen between wild-type and *hipA7* strains when *yfp* was fused to native *recN* and *rplA* in the chromosome (***B*** and ***C***).

Details. (*A−C*) Cultures were grown overnight to stationary phase (16 h), diluted 1,000-fold into fresh LB broth, or enriched for persisters by treatment for 10 h with 20 MIC ampicillin. Wild-type strains were concentrated 1,000-fold to normalize initial CFU. Samples were removed at the indicated times following dilution or enrichment for plating and CFU determination. Panel *A*: wild-type strain (0001) and *hipA7* mutant (0022); panel *B*: wild-type stain with *recN-yfp* (0141) and *hipA7*::*recN-yfp* (0140); panel *C*: wild-type strain with *rplA-yfp* (0182) and *hipA7::rplA-yfp* (0355). Data represent means ± SD (*n*=3).

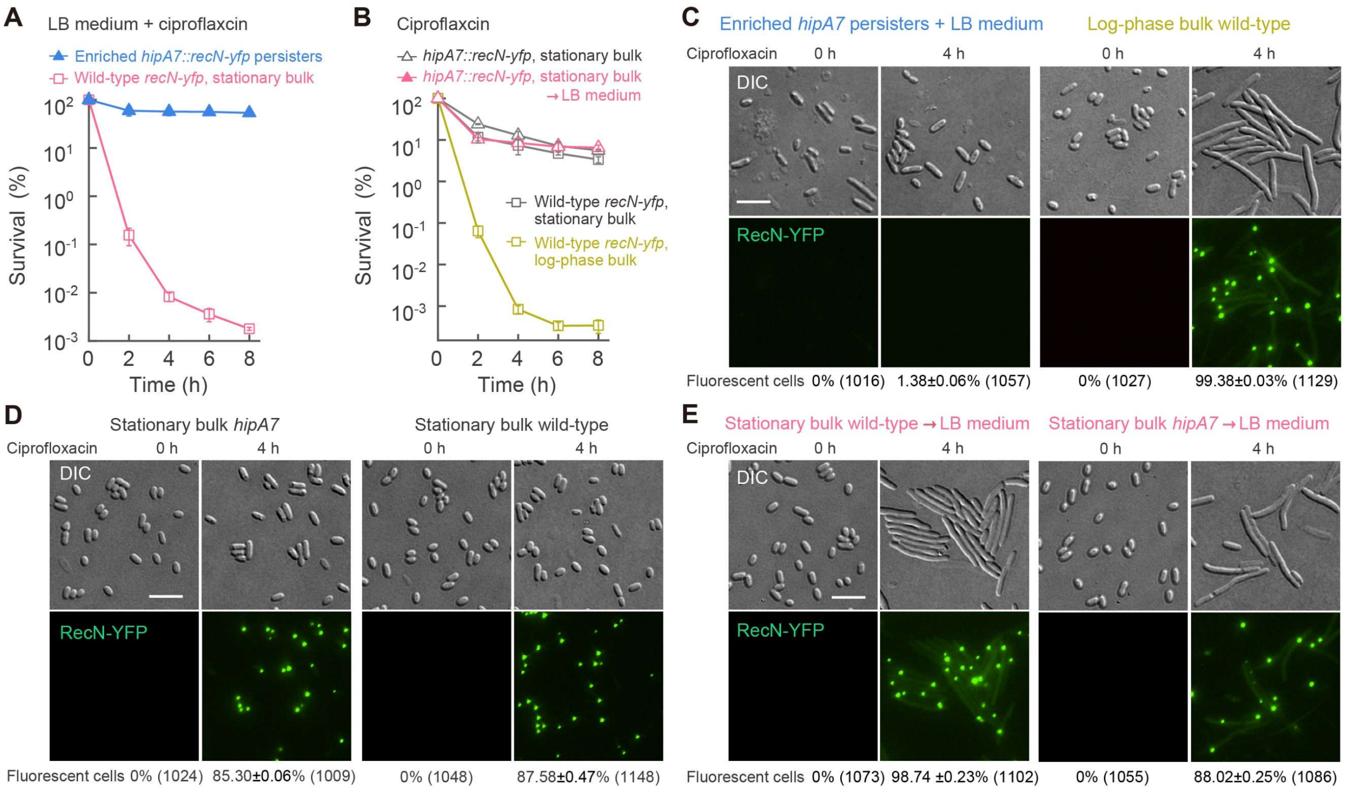

FIG S12. HipA7 persister cells enriched by ampicillin survive ciprofloxacin with little DNA breakage.

The DNA-breakage reporter gene (*recN-yfp*) had no effect on the behavior of either *hipA7* persisters or wild-type cells with respect survival during treatment with ciprofloxacin: enriched persisters were not killed in nutrient-rich medium, while bulk wild-type cells were (Panel ***A***). Survival of 10% was seen for bulk stationary-phase cultures of *hipA7* in the presence or absence of fresh nutrients and for the wild-type strain without fresh nutrients (***B***), as seen with their parent strains lacking *yfp* (Figs. 1A and 2A in the main text, and Fig. S3B). The enriched persister cells displayed a reduced level of double-strand DNA breaks, even after ciprofloxacin treatment for 4 h (***C***) or 6 h (Fig. 4A in the main text), with ~1% of the cells showing signs of DNA breakage in the form of RecN-YFP-mediated fluorescent foci. In contrast, DNA breakage signals were evident in 99-100% of cells in wild-type, log-phase cultures and in wild-type, stationary-phase cultures diluted into fresh medium containing ciprofloxacin (***C***, and Fig. 4A and 4C in the main text). For the major population of stationary-phase cultures treated with ciprofloxacin without dilution into fresh, nutrient-rich medium, approximately 85–88% of cells from both the *hipA7* and wild-type strains exhibited fluorescently marked DNA breakage foci (***D*** and ***E***). We conclude that the survival of *hipA7* persister cells correlates with the inability of fluoroquinolones to induce double-stranded DNA breaks.

Details. (*A*) Stationary-phase cultures of *hipA7* (0022) and *hipA7::recN-yfp* (0140) strains were treated with 20 MIC ampicillin for 10 h in fresh LB broth (20-fold dilution). After washing, cells were collected and incubated in fresh LB medium containing 10 MIC ciprofloxacin for 8 h (n=4, each). As controls, stationary-phase cultures of wild-type cells (strains 0001 and 0141; n=4, each) were diluted 20-fold into fresh medium with ciprofloxacin to assess killing. (*B*) Stationary-phase cultures of *recN-yfp*-bearing *hipA7* and wild-type strains (0140 and 0141; n=4,

each) were treated with 10 MIC ciprofloxacin in the absence of fresh medium. As a control, the wild-type strain (0141) was grown to log phase (OD_{600}=0.3), and then it was treated with ciprofloxacin. Survival for both panels *A* and *B* was assessed via standard LB agar. *n* denotes the number of independent biological replicates, and data represent the mean ± SD. (C) The strains and conditions (*n*=4) were as specified in panel *A*. After a 4-h treatment, cells of the *hipA7* and wild-type strains with *recN-yfp* present in the chromosome were visualized using fluorescence microscopy. (*D*) DNA damage in bulk stationary-phase cultures. The strains and conditions (*n*=4, in each case) were as in panel *B*. Cells treated for 4 h were imaged by microscopy. For panels *C* and *D*, the representative views are derived from 3 independent experiments yielding similar outcomes. Numbers in parentheses indicate total number of cells counted. Bar = 5 μm; DIC means differential interference contrast for viewing cell shape.

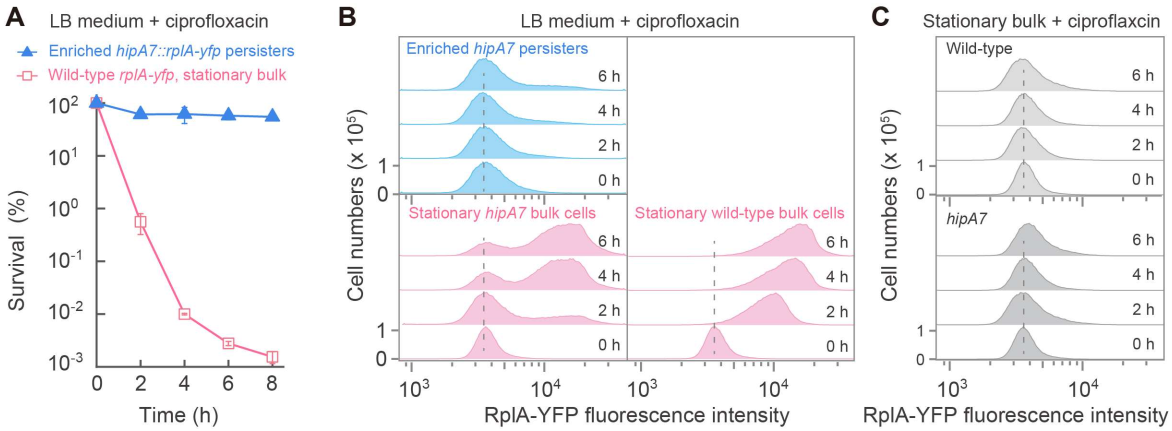

FIG S13. HipA7 persister cells show low-level translational activity.

When persister cells of a *hipA7* strain containing *rplA-yfp* in its chromosome were enriched using an ampicillin treatment, approximately 100% survived an 8-h treatment with 10 MIC ciprofloxacin (Panel ***A***). Exposure of ampicillin-enriched persister cells to 10 MIC ciprofloxacin in fresh, nutrient-rich medium led to little increase in cells exhibiting high-level RplA-YFP fluorescence (***B***, upper panel). However, when stationary-phase, wild-type cultures were incubated in fresh, rich medium containing ciprofloxacin, we observed that all cells accumulated high-level RplA-YFP fluorescence, almost six times above the mean fluorescence values (***B**,* right panel). Furthermore, when bulk cells of stationary-phase *hipA7* cultures were examined after ciprofloxacin treatment in rich medium, a subset of cells demonstrated lower RplA-YFP fluorescence levels compared to the bulk population (***B***, lower left panel), supporting the existence of a subpopulation with diminished translational activity. Suppression of translation was also seen in phenotypically tolerant cells, as RplA-YFP signals increased little when stationary-phase cultures of the *hipA7* mutant and wild-type strains were treated with ciprofloxacin (***C***). These findings support the notion that both persister and phenotypically tolerant cells survive antimicrobial treatment by low translation activity, with the later able to rapidly restore translation when shifted to nutrient-rich medium.

Details. (*A*) Persisters of *hipA7::rplA-yfp* strain (0355; *n*=4) were enriched by ampicillin and subjected to a ciprofloxacin treatment, as detailed in Fig. 4A in the main text, and Figs. S10 and S12A. In a comparison, stationary-phase wild-type cultures (strain 0182; *n*=3) were killed by ciprofloxacin in fresh medium following a 20-fold dilution. (*B*, *C*) During exposure to ciprofloxacin, as in *A*, RplA-YFP fluorescence in the enriched *hipA7::rplA-yfp* persisters (*n*=3) was measured using flow cytometry. Controls consisted of stationary-phase cultures of the *hipA7::rplA-yfp* and wild-type strains (*n*=3, each), diluted into fresh medium containing ciprofloxacin. Then the two strains (*n*=3, each) underwent ciprofloxacin treatment without dilution into fresh medium. Fluorescence was determined by flow cytometry. For panels *B* and *C*, the data are representative, as indicated by similar results with replicate experiments. *n* indicates the number of independent biological replicates.

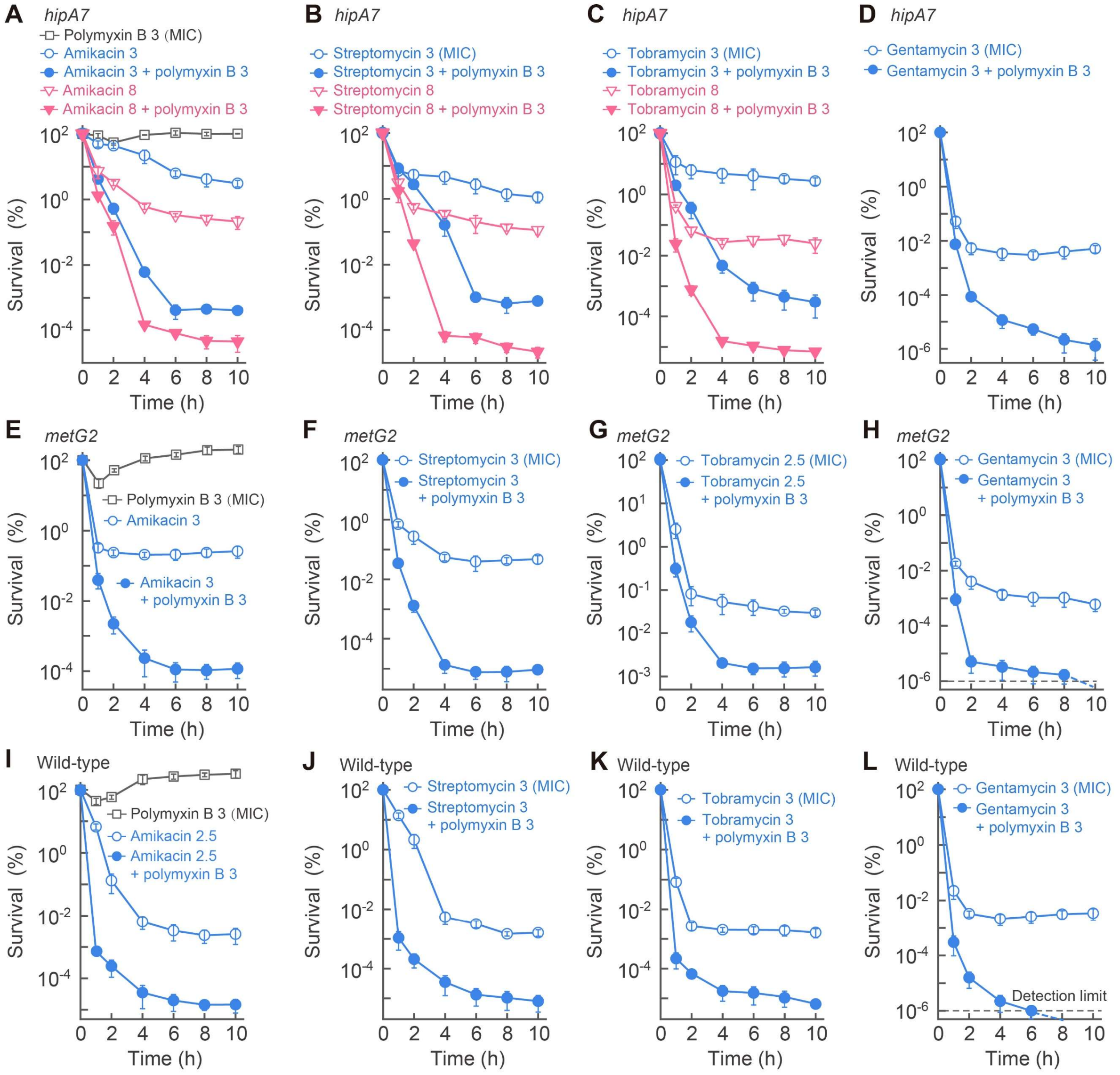

FIG S14. Combinations of polymyxin with various clinically used aminoglycosides kill persister and wild-type *E. coli* cells.

In addition to kanamycin, other members of the aminoglycoside family, when co-administered with polymyxin B, exhibited extensive killing of persister cells. When polymyxin B (3 MIC) was combined with amikacin (8 MIC), streptomycin (8 MIC), tobramycin (8 MIC), or gentamycin (3 MIC), survival of the *hipA7* persister mutant dropped to 10^{-5}–10^{-6} % (Panels ***A*–*C***). Lowering the concentration of the first three aminoglycosides to 3 MIC increased survival of the *hipA7* mutant to 10^{-3}–10^{-4} % (***A*–*C***), about a 10-fold increase compared to their 8 MIC doses; these values were still 10,000-fold lower than survival levels from treatment with each aminoglycoside alone. The antibiotic combinations also resulted in rapid and extensive death of the *metG2* mutant, with survival levels being 10^{-3}–10^{-6} % (***E*−*H***). These values were 10^{2}–10^{4}-fold lower than those observed with single aminoglycoside treatments and 10^{5}- to 10^{6}-fold lower than those from single polymyxin B treatments. Moreover, the persistent survival of the wild-type strain also dropped to 10^{-5}–10^{-6} %, marking a 100- to 1,000-fold decrease compared to treatment with

aminoglycoside alone (***I*–*L***). Thus, combinations of polymyxin B and various aminoglycosides at clinically attainable concentrations are highly effective in killing persister and susceptible *E. coli* cells.

Details. (*A–D*). Stationary-phase cultures of a *hipA7* mutant (strain 0022) were diluted 20-fold into fresh LB medium containing polymyxin B (3 MIC) and aminoglycosides at the concentrations indicated. Samples at the indicated times were washed twice by centrifugation prior to CFU determination. (*E-H*) The antibiotic combinations eliminated *metG2* persisters. The *metG2* mutant (0024) was treated as in panels *A−D*. The antibiotic concentrations were as indicated. (*I−L*) Eradication of susceptible and persister cells of the wild-type strain by the antibiotic combinations. The wild-type strain (0001) was treated at the conditions in panels *A−D.* In each case, the number of independent biological replicates was three. Data represent the mean ± SD.

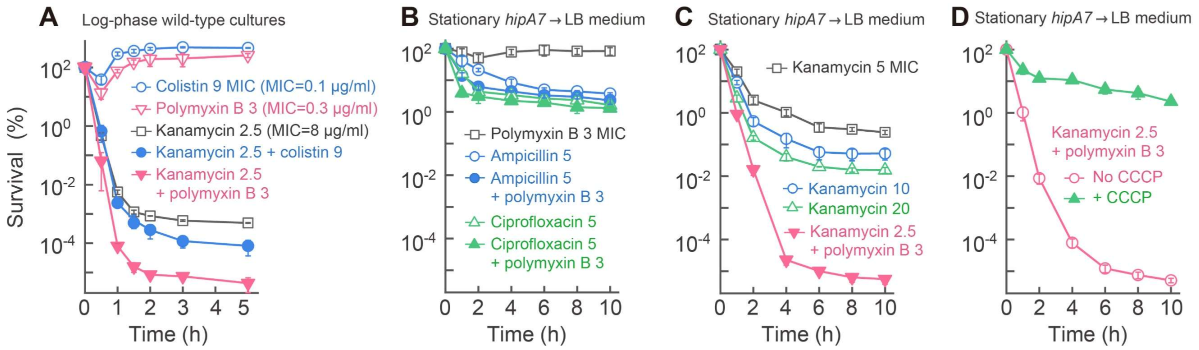

FIG S15. Eradication of persister cells by combination of polymyxin B with kanamycin but not with ampicillin or ciprofloxacin.

The persistence level of early log-phase, wild-type cultures was 0.001% following treatment with kanamycin at 2.5 MIC (20 µg/mL) (Panel ***A***). No death was seen during treatments with polymyxin B (3 MIC, 0.9 µg/mL) or colistin (polymyxin E; 9 MIC, 0.9 µg/mL) alone. When cultures were treated with kanamycin-polymyxin B and kanamycin-colistin combinations, survival rates dropped about 100- and 10-fold to 5×10^{-6} % and 1×10^{-4} %, respectively (***A***), indicating a greater potency of polymyxin B than colistin against persisters when in combination. The kanamycin-polymyxin B combination also reduced survival of *hipA7* persister cultures to 5×10^{-6} % (***C***). Surprisingly, the survival level of the *hipA7* mutant dropped only to 0.01% under exposure to the high kanamycin concentration of 160 µg/mL (20 MIC, 8-fold higher than peak serum concentration), a survival value that is 2,000-fold higher than that achieved with the combination therapy. Considering that 3 MIC polymyxin is non-lethal to *hipA7* persisters (***B***), the synergistic effect of kanamycin and polymyxin in eradicating persisters was quite evident. However, this potent persister-killing capacity was negated by the administration of 20 µg/mL of CCCP (***D***), a compound known to block proton motive force, which is required for aminoglycoside uptake (21, 22). In contrast to results with polymyxin-aminoglycoside combinations, combining 3 MIC polymyxin B with either 5 MIC ampicillin or ciprofloxacin produced persistent survival of a *hipA7* mutant that was comparable to survival observed from treatments using each individual antibiotic (***B***). These findings imply that the synergistic approach of combining polymyxin and aminoglycosides to eliminate persister cells does not apply broadly to other routinely employed antimicrobial classes.

Details. (*A*) Polymyxin plus kanamycin eliminates persisters in log-phase, wild-type cultures. Overnight cultures of the wild-type strain (0001; *n*=3) were diluted 200-fold into fresh LB medium and grown to $OD_{600} = 0.3$. Either polymyxin B (3 MIC) or colistin (9 MIC), kanamycin (2.5 MIC), or a combination of the two classes, was introduced into these cultures. Samples at the indicated times were washed twice followed by CFU measurement. (*B*) Stationary-phase cultures of a *hipA7* mutant (0022; *n*=3) were diluted 20-fold into fresh LB medium that contained either polymyxin B (3 MIC), ampicillin (5 MIC), ciprofloxacin (5 MIC), or a combination of polymyxin with either ampicillin or ciprofloxacin. (*C*) Elimination of *hipA7* persisters by a kanamycin-polymyxin B combination. The *hipA7* strain (0022, *n*=3) was treated with either kanamycin alone or kanamycin plus polymyxin B at conditions in Panel *A*. (*D*) Strain and conditions were as in Panel C, with 20 µg/mL CCCP added as indicated (*n*=3). *n* indicates the number of independent biological replicates. Data represent the mean ± SD.

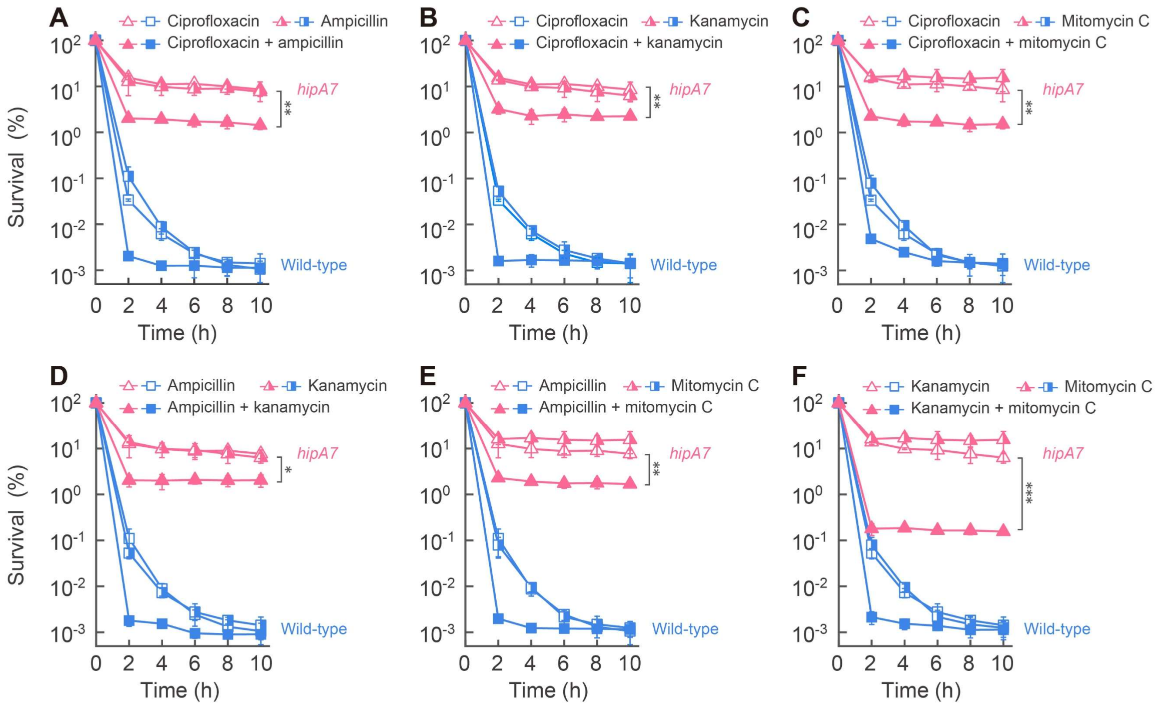

FIG S16. Effect of antibiotic combinations on survival of wild-type and *hipA7* cultures.

To examine cross-persistence among antibiotics with the *hipA7* mutant, stationary-phase cultures were diluted 20-fold into LB medium and treated with pairs of antibiotics. In general, combinations with the *hipA7* mutants (red symbols) were more lethal (by less than 10-fold) than the single compounds when ciprofloxacin was combined with ampicillin (***A***), kanamycin (***B***), or mitomycin C (***C***). Similar results were obtained when ampicillin was combined with kanamycin (***D***) or mitomycin C (***E***). We conclude that for these combinations there is a modest additivity of lethal action. The combination of kanamycin and mitomycin C was 50-times more lethal, indicating a stronger lethal synergy. With wild-type cells (blue symbols), combinations showed a more rapid killing than single drugs; for both single drugs and the combinations, killing was several orders of magnitude greater than with the *hipA7* mutant.

<u>Details</u>. Wild-type (strain 0001) and *hipA7* (strain 0022) cultures were grown in LB broth to stationary phase, and the cultures were diluted 20-fold into fresh LB containing the indicated antibiotic combinations. Antibiotic concentrations were as indicated in the figure panels. After treatment for 10 h, 300-μL samples were taken at the indicated times, washed twice with 1 mL saline, and resuspended in 300 μL saline. After serial dilution, percent survival was determined by plating, incubation, and colony counting. *n* equaled three independent biological replicates in all cases. Data represent the mean ± SD. *, $p<0.05$; **, $p<0.01$; ***, $p<0.001$.

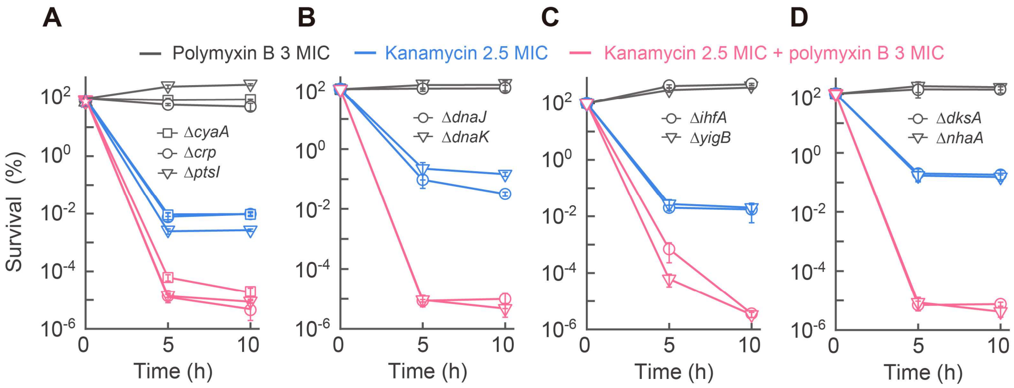

FIG S17. Combination of kanamycin and polymyxin B eradicates tolerance-associated mutants.

The combination of kanamycin (2.5 MIC) and polymyxin B (3 MIC) resulted in survival of 1×10^{-5} % for several mutants that exhibit various levels of antibiotic tolerance. The mutations tested included Δ*cyaA*, Δ*crp*, Δ*ptsI* (23), Δ*dnaJ*, Δ*dnaK*, Δ*dksA*, Δ*yigB*, Δ*ihfA* (24, 25), and Δ*nhaA* (26). By combining these results with the data using the *hipA7* and *metG2* persisters and wild-type cells (Fig. 5A–D in the main text), we conclude that combining a polymyxin with an aminoglycoside eradicates susceptible, tolerant, and persister cells of *E. coli*.

Details. (*A−D*) Overnight cultures of each strain were diluted 20-fold into fresh LB medium containing kanamycin (20 µg/mL, 2.5 MIC) and polymyxin B (0.9 µg/mL, 3 MIC). Samples at the indicated times were washed and plated for CFU determination. Three independent biological replicates were performed for each strain. Data represent the mean ± SD.

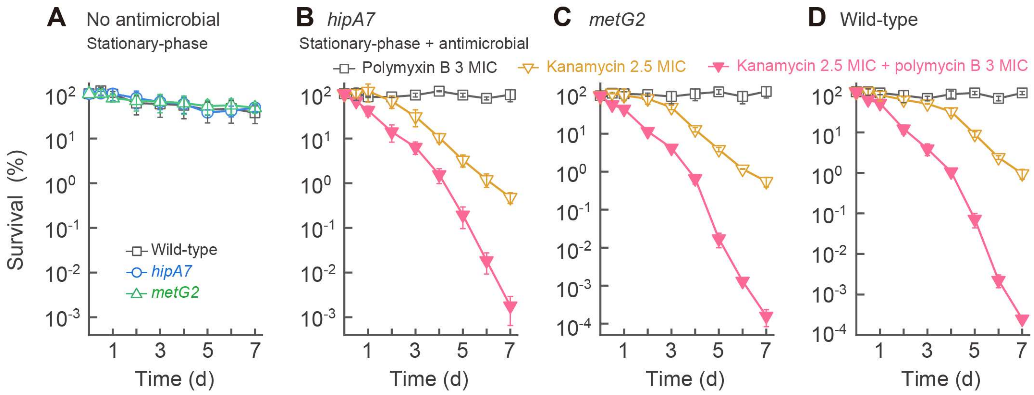

FIG S18. Long incubation periods allow a polymyxin-amikacin combination to kill stationary-phase, tolerant cultures in the absence of dilution into fresh nutrient medium.

E. coli cells maintained approximately 100% viability after 7 days in stationary phase (Panel ***A***). However, when stationary-phase cultures were treated with kanamycin (3 MIC) plus polymyxin B (3 MIC) for 7 days in the absence of dilution into rich medium, survival of the *hipA7*, *metG2* and wild-type strains dropped 5–6 orders of magnitude (***B−D***). The values were 10^3 to 10^4-fold lower than a treatment with kanamycin alone. Polymyxin B alone did not kill phenotypically tolerant cultures. Taken together with data using tolerance-associated mutants, the data lead to the conclusion that combinations of aminoglycosides and polymyxins have a high propensity for killing susceptible, tolerant, and persistent cells.

Details. (*A−D*) Stationary-phase cultures of *hipA7, metG2,* and wild-type strains (0022, 0024 and 0001) were treated with kanamycin (2.5 MIC), polymyxin B (3 MIC), or a combination of the two without addition of exogenous nutrients. In panel *A*, stationary-phase cultures in the absence of antimicrobials were used as controls. In each case, *n* equals 5 independent biological replicates. Data represent the mean ± SD.

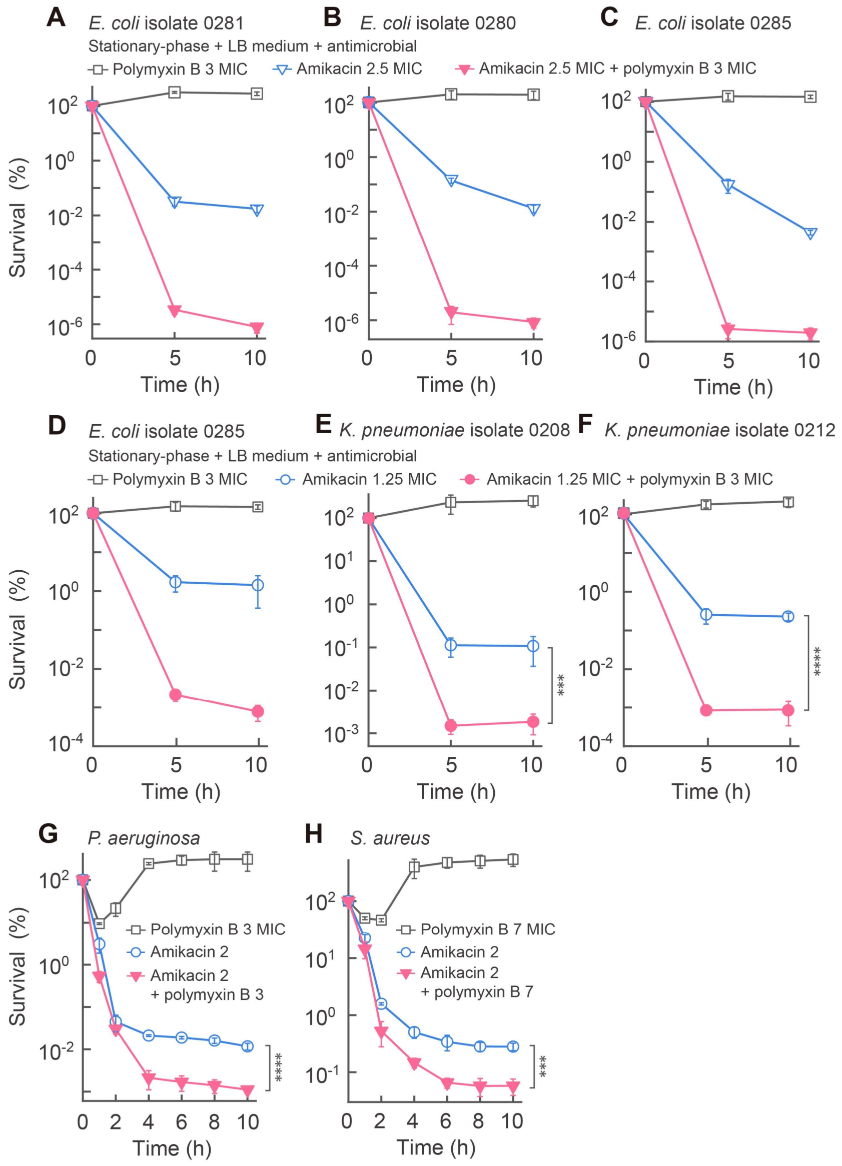

FIG S19. Polymyxin-amikacin combination reduces persistent survival of clinical isolates of *E. coli* and *K. pneumoniae* and a laboratory strain of *S. aureus*.

Polymyxin B (3 MIC, 0.9 µg/mL) alone did not kill clinical isolates of *E. coli* (Panels ***A−C***) or *K. pneumoniae* (***D*** and ***E***). Amikacin alone at 2.5 or 1.25 MIC dropped survival to 0.01-1%. The combination of polymyxin B and amikacin reduced percent survival of the *E. coli* isolates to 1×10^{-6}, 10^4-fold lower than the levels seen with amikacin alone. Reduction of amikacin to 1.25 MIC in combination with polymyxin B dropped survival levels of the two bacterial species to 0.001%, 100-1,000-fold lower than levels with amikacin alone (***C–E***). Taken with results of other clinical isolates (Fig. 5 E and F in the main text), we conclude that a combination of aminoglycoside and polymyxin synergistically potentiates eradication of clinical isolates of these bacterial pathogens.

Details. (*A−H*) Stationary-phase cultures of clinical *E. coli* (strains of 0280, 0281, and 0285; *n*=3, each) and *K. pneumoniae* isolates (strains of 0208 and 0212; *n*=3, each) were diluted 20-fold into fresh LB medium containing polymyxin B (3 MIC), amikacin (2.5 or 1.25 MIC) or both for each clinical isolate. Aliquots sampled at the

indicated times were washed twice and plated for CFU determination. *n* indicates the number of independent biological replicates. Data represent the mean ± SD. ***, $p<0.001$; ****, $p<0.0001$.

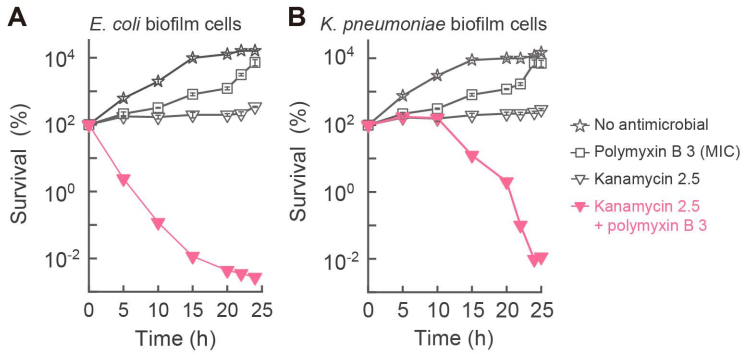

FIG S20. Killing biofilm bacteria by a combination of kanamycin and polymyxin B.

A biofilm model using a plastic culture dish showed that a polymyxin-aminoglycoside combination killed two bacterial species, *E. coli* and *K. pneumoniae*. Treatment with the individual compounds did not.

Details. Cultures *E. coli* (strain 0001, Panel *A*) and *K. pneumoniae* (strain 0066, Panel *B*) were grown overnight to stationary phase and diluted 10,000-fold. Aliquots of 1 mL were placed in each well of a 24-well plastic culture plate, and plates were incubated at 37 ºC for 40 h. Cells not attached to the walls of the wells were removed by washing, and LB medium was added containing kanamycin (2.5 MIC), polymyxin B (3 MIC), a combination of the two, or no treatment, as indicated in the figure (numbers are MICs). At the indicated times, wells were sonicated to dislodge wall-bound cells, which were then diluted and plated to determine CFU. Data represent the mean ± SD (*n*=4).

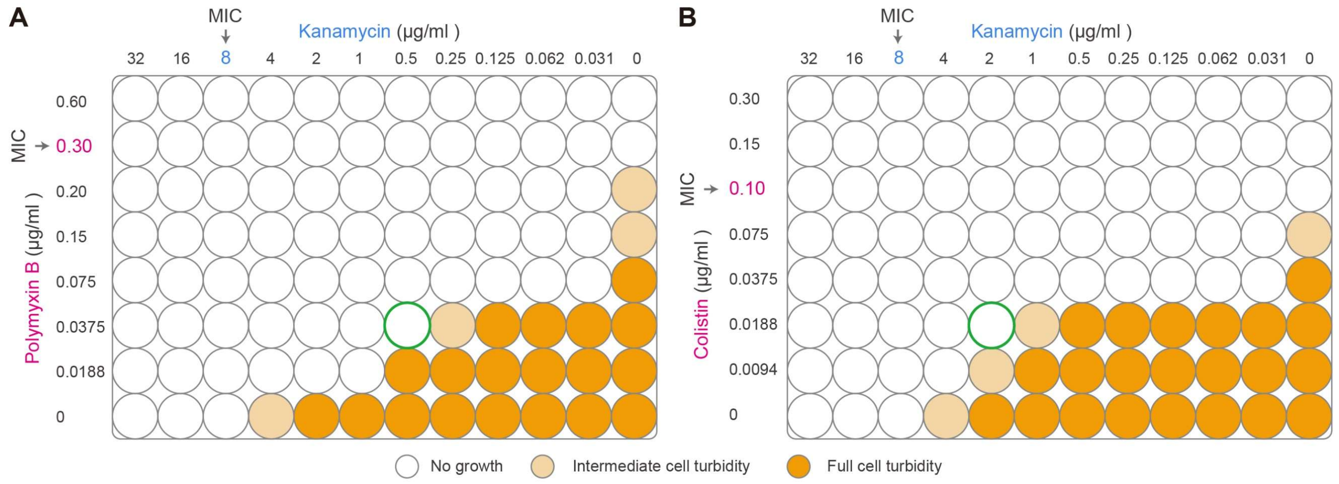

FIG S21. Bacteriostatic synergism between kanamycin and polymyxins.

The MICs for kanamycin, polymyxin B, and colistin with *E. coli* BW25113 were 8, 0.3, and 0.1 µg/mL, respectively (Panels ***A*** and ***B***; Table S2). As shown by a checkboard test, cell growth was inhibited when 0.5 µg/mL kanamycin and 0.0375 µg/mL polymyxin B were combined; the fractional inhibitory concentration index (FICI) value of the combination was 0.19 according to the FICI formula (2). Bacterial growth was also inhibited when 2 µg/mL kanamycin and 0.019 µg/mL colistin were combined; the corresponding FICI value was 0.44. Both FICI values were below 0.5, indicating that the two polymyxins in combination with kanamycin have synergistic effects.

<u>Details</u>. Early log-phase cultures of the wild-type strain (0001) were diluted to ~10^5 CFU/mL. The cultures were treated with kanamycin and polymyxin B or colistin at the indicated concentrations in each well using a 2-fold dilution series. Growth was determined by turbidity after incubation for 20–24 h. Three independent repeats showed the same result.

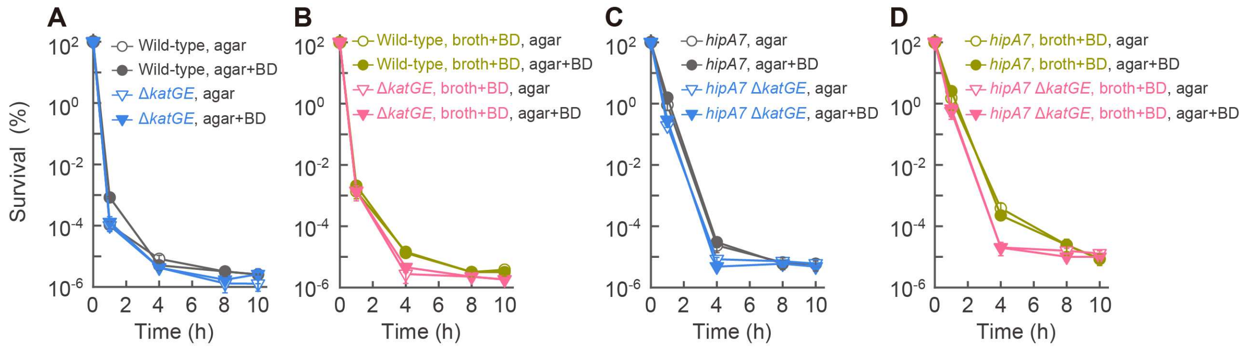

FIG S22. Eradication of persister cells by a polymyxin B-kanamycin combination is independent of ROS.

When stationary-phase cultures of the Δ*katGE* and wild-type strains were treated with 2.5 MIC kanamycin plus 3 MIC polymyxin B, survival dropped to the CFU detection limit (1×10^{-6} %). The introduction of anti-oxidants (bipyridyl plus DMSO) in both liquid cultures and in recovery agar failed to raise survival levels (Panels ***A*** and ***B***). In a similar situation, the *hipA7* and *hipA7* Δ*katGE* strains displayed little anti-oxidant protection or enhanced elimination, with persistence values at approximately 1×10^{-5} % (***C*** and ***D***). These observations, as well as finding 1) little ROS signal, 2) little quinolone-specific DNA damage, and 3) little increase in RplA-YFP-reported translational activity for *hipA7* persisters (see Fig. 3 E and F, and Fig. 4 A–F in the main text), lead to the conclusion that the eradication of susceptible and persister cells by the polymyxin B-kanamycin combination is largely independent of ROS action.

Details. (*A*−*D*) Stationary-phase cultures of the Δ*katGE* and wild-type strains (0042 and 0001; *n*=4, each) were diluted 20-fold into fresh LB medium containing 0.9 µg/mL polymyxin B and 20 µg/mL kanamycin. In parallel, 0.3 mM bipyridyl plus 5% DMSO were present in both liquid cultures and recovery agar. After washing, CFU was assessed by plating. To address death of *hipA7* and *hipA7* Δ*katGE* cells being associated with little ROS during treatment with the combination of polymyxin B plus kanamycin, we treated *hipA7* and *hipA7* Δ*katGE* (0022 and 0354; *n*=4, each) cultures as in panel *A*. *n* indicates the number of independent biological replicates. Data represent the mean ± SD.

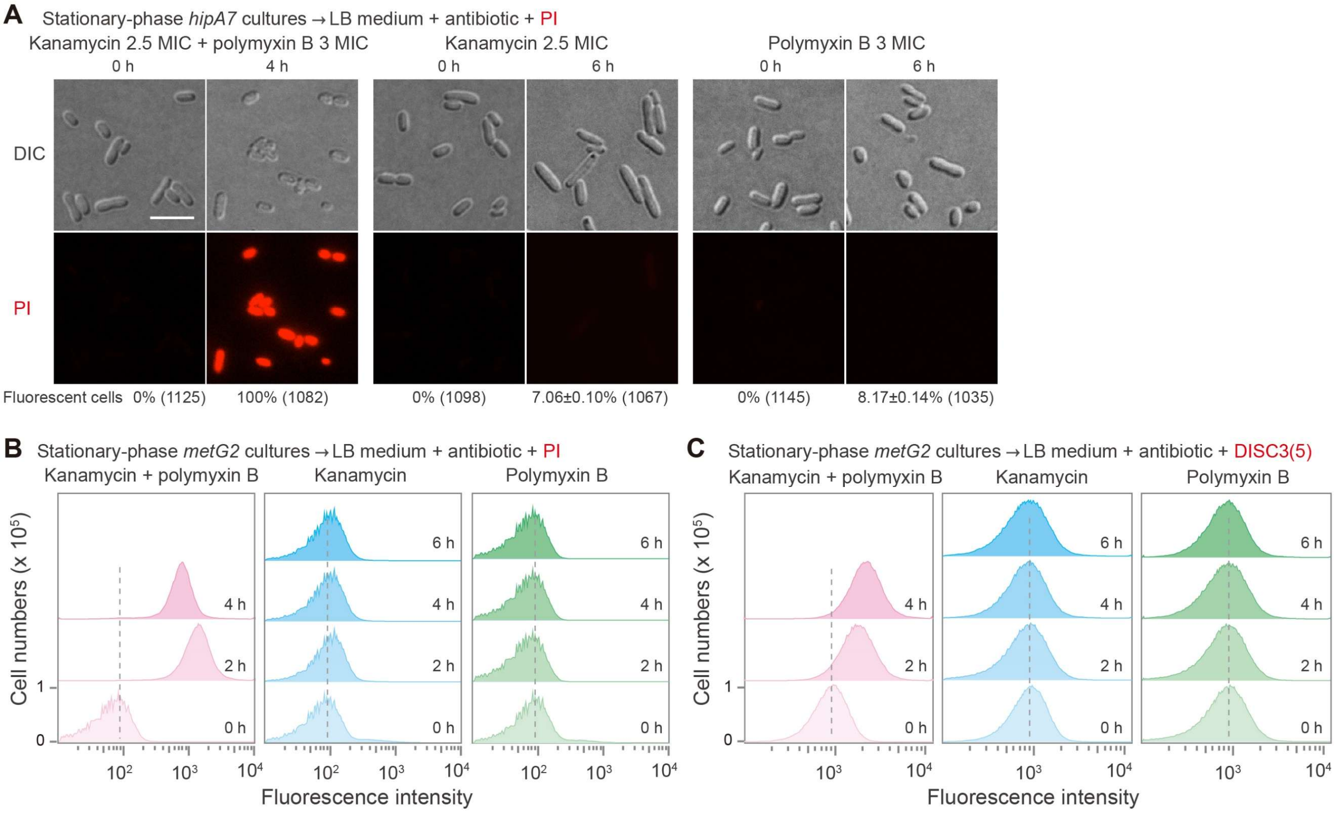

FIG S23. Aminoglycoside-polymyxin combination damages membranes of persister cells.

When stationary-phase cultures of the *hipA7* mutant were diluted into rich medium and exposed to a combination of kanamycin (2.5 MIC) and polymyxin B (3 MIC) for 4 h, cell death was extensive: 100% of cells died as evidenced by propidium iodide (PI) staining seen by microscopy (Panel ***A***). This outcome was consistent with survival of 1×10^{-7} % as quantified by CFU determination (Fig. 5C in the main text). In contrast, treatment using either kanamycin or polymyxin B alone for 6 h induced propidium iodide (PI) fluorescence in only a modest population of cells (7–8%) (***A***). Correspondingly, the combined antibiotic regimen showed membrane damage with the high-level, persistent *metG2* mutant, as denoted by fluorescence labeling with propidium iodide (***B***) and DiSC3(5) (***C***). Neither kanamycin nor polymyxin B, when administered separately, caused a significant increase in fluorescence. Overall, the data from bulk cultures underscores the potent synergistic action of aminoglycosides and polymyxins in rupturing cell membranes and killing persister cells.

Details. (*A*) Stationary-phase cultures of *hipA7* mutant (0022; *n*=4) were diluted 20-fold into fresh LB broth containing kanamycin (2.5 MIC), polymyxin B (MIC), and a combination of the two. PI was present at the onset of killing. After washing, cells were viewed by microscopy. (*B*) To address PI-labeled death of *metG2* bulk cells following treatment with kanamycin and polymyxin, *metG2* cultures (0024; *n*=3) were treated as in panel *A*. Fluorescence was assessed via flow cytometry. (*C*) Membrane damage occurred in *metG2* bulk cells as a result of kanamycin-polymyxin combination treatment. The *metG2* cultures (*n*=3) were treated as per the procedure in panel *A*, with DiSC3(5) substituting for PI. Data shown are representative of three or four independent biological replicates that yielded similar results.

Table S1. Bacterial strains used in the study.

Strain	Relevant genotype	Source, derivation, or reference [a, b, c]
0001	Wild-type *E. coli* BW25113, F-, Δ(*araD-araB)567,* Δ*lacZ4787(::rrnB-3), λ-, rph-1,* Δ*(rhaD-rhaB)568, hsdR514*	CGSC 7636
0020	F-, *Δ(argF-lac)169, gal-490, Δ(modF-ybhJ)803, λ[cI857 Δ(cro-bioA)], recN796-yfp(::cat), IN(rrnD-rrnE)1, rph-1*, Cm^R	CGSC 12775
0021	*hipA7::tet,* Tet^R, *E. coli* BW25113	Stock in this lab
0022	*hipA7*, *E. coli* BW25113	Remove marker from 0021
0024	*metG2,* deleted the last 7-aa residues at C-terminus, *E. coli* MG1655	Dr. Saeed Tavazoie[c] (27)
0042	Δ*katGE*, *E. coli* BW25113	Stock in this lab
0063	Δ*ptsI*, *E. coli* BW25113	Stock in this lab
0064	Δ*cyaA::kan,* Kan^R, *E. coli* BW25113	Stock in this lab
0065	Δ*crp::kan,* Kan^R, *E. coli* BW25113	Stock in this lab
0066	*Klebsiella pneumoniae* ATCC43816	Stock in this lab
0067	*Staphylococcus aureus* RN4220	Stock in this lab
0075	pCas9 plasmid in *E. coli* DH5α, Kan^R, *repA101* temperature-sensitive replicon (30 °C), λ_{Red} recombinase genes	Dr. Sheng Yang[c] (3)
0076	pTargetF-gRNA plasmid in *E. coli* DH5α, $Spec^R$	Dr. Sheng Yang
0077	pCP20 plasmid in *E. coli* DH5α, yeast FLP recombinase gene, temperature-sensitive replicon (30 °C), Amp^R, Cm^R	CGSC 7629
0140	*hipA7::recN-yfp::cat,* Cm^R, *E. coli* BW25113	0021 x P1 (0020)
0141	Wild-type, *recN-yfp::cat,* Cm^R, *E. coli* BW25113	0001 x P1 (0020)
0182	Wild-type, *rplA-yfp::cat*, Cm^R, *E. coli* BW25113	Stock in this lab, originally constructed by the λ_{Red} system
0208	Clinical *Klebsiella pneumoniae*, carbapenem-resistant, isolate from human respiratory tract infection	Dr. Haifang Zhang[c]
0210	Clinical *Klebsiella pneumoniae*, carbapenem-resistant, isolate from human bloodstream infection	Dr. Haifang Zhang
0212	Clinical *Klebsiella pneumoniae*, carbapenem-resistant, isolate from human respiratory tract infection	Dr. Haifang Zhang
0227	*Pseudomonas aeruginosa* ATCC27853	Dr. Haifang Zhang
0280	Clinical *E. coli*, isolate from human urinary infection	Dr. Zhemin Zhou[c]
0281	Clinical *E. coli*, isolate from human urinary infection	Dr. Zhemin Zhou
0284	Clinical *E. coli*, isolate from human urinary infection	Dr. Zhemin Zhou
0285	Clinical *E. coli*, isolate from human urinary infection	Dr. Zhemin Zhou
0318	Δ*yigB*, *E. coli* BW25113	Marker removal of CGSC 10751 by pCP20 plasmid
0319	Δ*ihfA*, *E. coli* BW25113	Marker removal of CGSC 9441 by pCP20 plasmid
0320	Δ*dksA*, *E. coli* BW25113	Marker removal of CGSC 8412 by pCP20 plasmid
0321	Δ*dnaJ*, *E. coli* BW25113	Marker removal of CGSC 8343 by pCP20 plasmid
0322	Δ*dnaK*, *E. coli* BW25113	Marker removal of CGSC 8342 by pCP20 plasmid
0342	Δ*cyaA*, *E. coli* BW25113	Marker removal of 0064 by pCP20 plasmid
0343	Δ*crp*, *E. coli* BW25113	Marker removal of 0065 by pCP20 plasmid
0354	*hipA7* Δ*katGE*, *E. coli* BW25113	Constructed by CRISPR
0355	*hipA7::rplA-yfp::cat,* Cm^R, *E. coli* BW25113	0021 x P1 (0182)
0394	Δ*nhaA*, *E. coli* BW25113	Marker removal of CGSC 8344 by pCP20 plasmid

Strain	Relevant genotype	Source, derivation, or reference [a, b, c]
0696	Δ*ahpCF*, *E. coli* BW25113	Constructed by CRISPR
0697	*hipA7* Δ*ahpCF*, *E. coli* BW25113	Constructed by CRISPR
0699	pACYC184 plasmid in *E. coli* BW25113, Cm^R	Stock in this lab
0701	pACYC-*katE* recombinant plasmid in Δ*katGE*, *E. coli* BW25113, Cm^R	Stock in this lab
0702	pACYC184 plasmid in Δ*katGE*, *E. coli* BW25113, Cm^R	Stock in this lab
0705	pACYC184 plasmid in *hipA7*, *E. coli* BW25113, Cm^R	Stock in this lab
0707	pACYC-*katE* recombinant plasmid in *hipA7* Δ*katGE*, *E. coli* BW25113, Cm^R	Stock in this lab
0708	pACYC184 plasmid in *hipA7* Δ*katGE*, *E. coli* BW25113, Cm^R	Stock in this lab

[a] x P1 indicates strain constructed by bacteriophage P1-mediated transduction, with phage prepared in the indicated strain.
[b] CGSC, Coli Genetic Stock Center.
[c] Dr. Saeed Tavazoie is located at Columbia University, USA. Dr. Sheng Yang is located at Shanghai Institutes for Biological Sciences, Chinese Academy of Sciences. Drs. Haifang Zhang and Zhemin Zhou are located at Soochow University, China.

Table S2. Susceptibility (MIC) of strains to antimicrobials in the study.

Strain	Genotype	MIC (µg/mL)												
		Ciprofloxacin	Ampicillin	Polymyxin B	Colistin	Kanamycin	Amikacin	Streptomycin	Gentamycin	Tobramycin	Mitomycin C	Oxolinic acid	DMSO	Bipyridyl
0001	Wild-type	0.03	6	0.3	0.1	8	2	4	4.8	2	1.2	0.6	15% v/v	0.75 mM
0022	*hipA7*	0.03	6	0.3	0.1	8	2	4	4.8	2	1.2	0.6	15% v/v	0.75 mM
0024	*metG2*	0.03	6	0.3	0.1	8	2	4	4.8	2	1.2	0.6	15% v/v	0.75 mM
0042	Δ*katGE*	0.03	6	ND	ND	ND	ND	ND	ND	ND	ND	ND	ND	ND
0354	*hipA7* Δ*katGE*	0.03	6	ND	ND	ND	ND	ND	ND	ND	ND	ND	ND	ND
0141	Wild-type *recN-yfp*	0.03	6	ND	ND	ND	ND	ND	ND	ND	ND	ND	ND	ND
0140	*hipA7::recN-yfp*	0.03	6	ND	ND	ND	ND	ND	ND	ND	ND	ND	ND	ND
0182	Wild-type *rplA-yfp*	0.03	6	ND	ND	ND	ND	ND	ND	ND	ND	ND	ND	ND
0355	*hipA7::rplA-yfp*	0.03	6	ND	ND	ND	ND	ND	ND	ND	ND	ND	ND	ND
0063	Δ*ptsI*	0.03	6	0.3	ND	8	ND	ND	ND	ND	ND	ND	ND	ND
0318	Δ*yigB*	0.03	6	0.3	ND	8	ND	ND	ND	ND	ND	ND	ND	ND
0319	Δ*ihfA*	0.03	6	0.3	ND	8	ND	ND	ND	ND	ND	ND	ND	ND
0320	Δ*dksA*	0.03	6	0.3	ND	8	ND	ND	ND	ND	ND	ND	ND	ND
0321	Δ*dnaJ*	0.03	6	0.3	ND	8	ND	ND	ND	ND	ND	ND	ND	ND
0322	Δ*dnaK*	0.03	6	0.3	ND	8	ND	ND	ND	ND	ND	ND	ND	ND
0342	Δ*cyaA*	0.03	6	0.3	ND	8	ND	ND	ND	ND	ND	ND	ND	ND
0343	Δ*crp*	0.03	6	0.3	ND	8	ND	ND	ND	ND	ND	ND	ND	ND
0394	Δ*nhaA*	0.03	6	0.3	ND	8	ND	ND	ND	ND	ND	ND	ND	ND
0208	*K. pneumoniae* isolate	ND	ND	0.3	ND	ND	12.8	ND	ND	ND	ND	ND	ND	ND
0210	*K. pneumoniae* isolate	ND	ND	0.3	ND	ND	12.8	ND	ND	ND	ND	ND	ND	ND
0212	*K. pneumoniae* isolate	ND	ND	0.3	ND	ND	12.8	ND	ND	ND	ND	ND	ND	ND
0280	*E. coli* isolate	ND	ND	0.3	ND	ND	16	ND	ND	ND	ND	ND	ND	ND
0281	*E. coli* isolate	ND	ND	0.3	ND	ND	16	ND	ND	ND	ND	ND	ND	ND
0284	*E. coli* isolate	ND	ND	0.3	ND	ND	16	ND	ND	ND	ND	ND	ND	ND
0285	*E. coli* isolate	ND	ND	0.3	ND	ND	16	ND	ND	ND	ND	ND	ND	ND
0066	*K. pneumoniae*	ND	ND	0.3	ND	8	ND	ND	ND	ND	ND	ND	ND	ND
0067	*S. aureus*	ND	ND	0.3	0.6	12	8	ND	ND	ND	ND	ND	ND	ND
0227	*P. aeruginosa*	ND	ND	0.7	0.8	>64	6	ND	ND	ND	ND	ND	ND	ND

		MIC (µg/mL)												
Strain	Genotype	Ciprofloxacin	Ampicillin	Polymyxin B	Colistin	Kanamycin	Amikacin	Streptomycin	Gentamycin	Tobramycin	Mitomycin C	Oxolinic acid	DMSO	Bipyridyl
0696	Δ*ahpCF*	0.03	6	ND	ND	ND	ND	ND	ND	ND	ND	ND	ND	ND
0697	*hipA7* Δ*ahpCF*	0.03	6	ND	ND	ND	ND	ND	ND	ND	ND	ND	ND	ND
0699	Wild-type + pACYC184	0.03	6	ND	ND	ND	ND	ND	ND	ND	ND	ND	ND	ND
0701	Δ*katGE* + pACYC-*katE*	0.03	6	ND	ND	ND	ND	ND	ND	ND	ND	ND	ND	ND
0702	Δ*katGE* + pACYC184	0.03	6	ND	ND	ND	ND	ND	ND	ND	ND	ND	ND	ND
0705	*hipA7* + pACYC184	0.03	6	ND	ND	ND	ND	ND	ND	ND	ND	ND	ND	ND
0707	*hipA7* Δ*katGE* + pACYC-*katE*	0.03	6	ND	ND	ND	ND	ND	ND	ND	ND	ND	ND	ND
0708	*hipA7* Δ*katGE* + pACYC184	0.03	6	ND	ND	ND	ND	ND	ND	ND	ND	ND	ND	ND

ND, not determined.

Table S3. Primers used in the study.

No.	Primer[a]	Sequence of primers (5′→3′)	Description[b]
1	Check-*metG2*-F	GCAGGTTGAAGCACTGGTGGAA	Forward (F) and reverse (R) primers for verification of the *metG2* mutation by sequencing.
2	Check-*metG2*-R	GAATTATCCTTCGCTTGCTCCA	
3	Check-*hipA7*-F	TAACGACTACCAGAAAAGCAC	Two primers for verification of the *hipA7* single-site mutation by sequencing.
4	Check-*hipA7*-R	TGGTAATGCCTAAACTTGTC	
5	*katG*-sgRNA-F	GGTACTAGTttgaagaacaaggtttaggtGTTTTAGAG CTAGAAATAGCAAGTT	Two primers for amplification of an sgRNA template with an N20 sequence (lowercase font) targeting *katG*. *katG*-sgRNA cloned into the pTargetF vector.
6	sgRNA-R	TGATGGAGCTGCACATGAAC	
7	*katG*-UF	GGGTTCGACCTGGTTTAT	Four primers used for cloning two homologous arms upstream (U) and downstream (D) of the open reading frames (ORF) of *katG*. The fusion fragment of the two arms and the pTargetF-*katG*-sgRNA were used to knockout the chromosomal *katG* ORF in the presence of Cas9. The *katG*-UF and -DR primers were also used for verification of the Δ*katG* deletion by sequencing.
8	*katG*-UR	AGAACGCCACGGATGCGATAGTTG	
9	*katG*-DF	CCGTGGCGTTCTTTACCAGCGTATAGTG	
10	*katG*-DR	GGGCAATGGCTAAGGTGTAT	
11	*katE*-sgRNA-F	GGTACTAGTaatggtgctcaatcgcaaccGTTTTAGA GCTAGAAATAGCAAGTT	This primer combined sgRNA-R was used for amplification of an sgRNA template with an N20 sequence (lowercase font) targeting *katE*. *katE*-sgRNA cloned into the pTargetF vector.
12	*katE*-UF	CTCTCCCATCAGTACAAACG	Four primers used for cloning two homologous arms upstream (U) and downstream (D) of the open reading frames (ORF) of *katE*. The fusion fragment of the two arms and the pTargetF-*katE*-sgRNA were used to knockout the chromosomal *katE* ORF in the presence of Cas9. The *katE*-UF and -DR primers were also used for verification of the Δ*katE* deletion by sequencing.
13	*katE*-UR	ATTTGAGGCGGATTACTGAAAGGGCCGCT	
14	*katE*-DF	CTTTCAGTAATCCGCCTCAAATGATTTACAT	
15	*katE*-DR	GCTTAACGGAGCTGGATG	
16	*ahpCF*-sgRNA-F	GGTACTAGTcctggatgcagcttgcatcaGTTTTAGAG CTAGAAATAGCAAGTT	This primer combined sgRNA-R was used for amplification of an sgRNA template with an N20 sequence (lowercase font) targeting *ahpCF*. *ahpCF*-sgRNA cloned into the pTargetF vector.
17	*ahpCF*-UF	GGCATAACCTATCACTGTCA	Four primers used for cloning two homologous arms upstream (U) and downstream (D) of the open reading frames (ORF) of *ahpCF*. The fusion fragment of the two arms and the pTargetF-*ahpCF*-sgRNA were used to knockout the chromosomal *ahpCF* ORF in the presence of Cas9. The *ahpCF*-UF and -DR primers were also used for verification of the Δ*ahpCF* deletion by sequencing.
18	*ahpCF*-UR	CTTACTTCTTCCTATACTTCCTCCGTGTTTT	
19	*ahpCF*-DF	GAGGAAGTATAGGAAGAAGTAAGATTCACCT G	
20	*ahpCF*-DR	GATGGTCAGCCACTTCAC	
21	Check-*rplA-yfp*-F	TGGTGCCGACATTGAAGC	Two primers for verification of the *rplA-yfp* fusion by sequencing.
22	Check-*rplA-yfp*-R	CTCCTCCAAACGGAGAGAGC	
23	Check-*recN-yfp*-F	CGGCTGGTCAAAAAAATTAT	Two primers for verification of the *recN-yfp* fusion by sequencing.
24	Check-*recN-yfp*-R	TCAGATAGTTCCCCTGGTTG	
25	*cyaA*-sgRNA-F	GGTACTAGTgacctttatttatgtgccgcGTTTTAGAGC TAGAAATAGCAAGTT	This primer combined sgRNA-R was used for amplification of an sgRNA template with an N20 sequence (lowercase font) targeting *cyaA*. *cyaA*-sgRNA cloned into the pTargetF vector.
26	*cyaA*-UF	CCGTCATTATCATCCGTG	Four primers used for cloning two homologous arms upstream (U) and downstream (D) of the open reading frames (ORF) of *cyaA*. The fusion fragment of the two arms and the pTargetF-*cyaA*-sgRNA were used to knockout the chromosomal *cyaA* ORF in the presence of Cas9. The *cyaA*-UF and -DR primers were also used for verification of the Δ*cyaA* deletion by sequencing.
27	*cyaA*-UR	GCACGTTCAGACGTATCGCCTGATTTG	
28	*cyaA*-DF	CGATACGTCTGAACGTGCCGGAAAGC	
29	*cyaA*-DR	TATACTGCCGCCATCACT	
30	*crp*-sgRNA-F	GGTACTAGTcaagtcacttcagagaaagtGTTTTAGA GCTAGAAATAGCAAGTT	This primer combined sgRNA-R was used for amplification of an sgRNA template with an N20 sequence (lowercase font) targeting *crp*. *crp*-sgRNA cloned into the pTargetF vector.

No.	Primer[a]	Sequence of primers (5′→3′)	Description[b]
31	*crp*-UF	GCTTTATCGCCTGAGTTG	Four primers used for cloning two homologous arms upstream (U) and downstream (D) of the open reading frames (ORF) of *crp*. The fusion fragment of the two arms and the pTargetF-*crp*-sgRNA were used to knockout the chromosome *crp* ORF in the presence of Cas9. The *crp*-UF and -DR primers were also used for verification of the Δ*crp* deletion by sequencing.
32	*crp*-UR	CGACGGGAGCGCGGTTATCCTCTGTT	
33	*crp*-DF	TAACCGCGCTCCCGTCGGAGTGGCGC	
34	*crp*-DR	TCAGCAGTGACGCCAAGT	
35	*ptsI*-sgRNA-F	GGTACTAGTctgccagctattacgctggaGTTTTAGAG CTAGAAATAGCAAGTT	This primer combined sgRNA-R was used for amplification of an sgRNA template with an N20 sequence (lowercase font) targeting *ptsI*. *ptsI*-sgRNA cloned into the pTargetF vector.
36	*pstI*-UF	AACAATACAGGCTAAAGTCG	Four primers used for cloning two homologous arms upstream (U) and downstream (D) of the open reading frames (ORF) of *ptsI*. The fusion fragment of the two arms and the pTargetF-*ptsI*-sgRNA were used to knockout the chromosomal *ptsI* ORF in the presence of Cas9. The *ptsI*-UF and -DR primers were also used for verification of the Δ*ptsI* deletion by sequencing.
37	*pstI*-UR	ATCTCGTGGAAACCCTACCTTACTTGTGAC	
38	*pstI*-DF	AGGTAGGGTTTCCACGAGATGCGGCCCAAT	
39	*pstI*-DR	TTCAGTTCAACGGTGTCG	
40	Check-*dnaK*-F	TTGTCCTGCCATATCGCG	Two primers for verification of the Δ*dnaK* deletion by sequencing.
41	Check-*dnaK*-R	TTCACGCTCTTCCGCTGT	
42	Check-*yigB*-F	AAACGCTTTGCCGAATGG	Two primers for verification of the Δ*yigB* deletion by sequencing.
43	Check-*yigB*-R	TTCCACGCTCATCAACCAG	
44	Check-*ihfA*-F	ATCCGTTCTGCTGAAGTGTC	Two primers for verification of the Δ*ihfA* deletion by sequencing.
45	Check-*ihfA*-R	ATAAGAGCCTCGCCATAA	
46	Check-*dksA*-F	CGTGATGGAACGGCTGTAA	Two primers for verification of the Δ*dksA* deletion by sequencing.
47	Check-*dksA*-R	TGCTGAAGGCATGGCTCT	
48	Check-*dnaJ*-F	GACGCTGAATTTGAAGAAGT	Two primers for verification of the Δ*dnaJ* deletion by sequencing.
49	Check-*dnaJ*-R	TCTTCAACGCACCCTATTT	
50	Check-*nhaA*-F	CGCCGACTGACAACAAAT	Two primers for verification of the Δ*nhaA* deletion by sequencing.
51	Check-*nhaA*-R	AAGGGAGCCGTTTATGGC	
52	pACYC-*katE*-F	GGTACTAGTTACTGGCTTCACTAAACGCA	Four primers for amplifying wild-type *katE* with its native promoter and a Cm^R-p15Aori fragment of the low-copy plasmid pACYC184 to construct pACYC-*katE* that is used for catalase complementation. The underlines indicate the *Spe*I and *Xho*I digestion sites, respectively.
53	pACYC-*katE*-R	GGTCTCGAGCATCAGGCAGGAATTTTGTC	
54	pACYC-F	GGTACTAGTCTGCTGGCTACCCTGTGG	
55	pACYC-R	GGTCTCGAGTTAACTGTGATAAACTACCGCA	
56	Check-*katE*-F	TCAAATGTAGCACCTGAAGTC	Two primers for verification of the inserted sequence of *katE* in pACYC-*katE* by sequencing.
57	Check-*katE*-R	GATCATTTATTCTGCCTCCC	

[a] Capital letters of F and R in primer names indicate forward and reverse primers for PCR amplification, respectively; check means primers used for mutation verification by sequencing; capital letters U and D indicate primers used for PCR amplification of homologous arms upstream and downstream of a specific gene for mutation, respectively.

[b] N20 indicates a 20-nt fragment complementary to a target DNA sequence preceding a protospacer adjacent motif, generating a guide RNA (gRNA) for Cas9-mediated cleavage.

Supplementary References

1. Thomason LC, Costantino N, Court DL. 2007. *E. coli* genome manipulation by P1 transduction. Curr Protoc Mol Biol Chapter 1:Unit 1 17.
2. Tyers M, Wright GD. 2019. Drug combinations: a strategy to extend the life of antibiotics in the 21st century. Nat Rev Microbiol 17:141-155.
3. Jiang Y, Chen B, Duan C, Sun B, Yang J, Yang S. 2015. Multigene editing in the *Escherichia coli* genome via the CRISPR-Cas9 system. Appl Environ Microbiol 81:2506-14.
4. Tong Y, Jorgensen TS, Whitford CM, Weber T, Lee SY. 2021. A versatile genetic engineering toolkit for *E. coli* based on CRISPR-prime editing. Nat Commun 12:5206.
5. Brandt R, Keston AS. 1965. Synthesis of diacetyldichlorofluorescin: a stable reagent for fluorometric analysis. Anal Biochem 11:6-9.
6. Hong Y, Li L, Luan G, Drlica K, Zhao X. 2017. Contribution of reactive oxygen species to thymineless death in *Escherichia coli*. Nat Microbiol 2:1667-75.
7. Hong Y, Zeng J, Wang X, Drlica K, Zhao X. 2019. Post-stress bacterial cell death mediated by reactive oxygen species. Proc Natl Acad Sci U S A 116:10064-71.
8. Dwyer DJ, Belenky PA, Yang JH, MacDonald IC, Martell JD, Takahashi N, Chan CTY, Lobritz MA, Braff D, Schwarz EG, Ye JD, Pati M, Vercruysse M, Ralifo PS, Allison KR, Khalil AS, Ting AY, Walker GC, Collins JJ. 2014. Antibiotics induce redox-related physiological alterations as part of their lethality. Proc Natl Acad Sci U S A 111:E2100-9.
9. Sanchez H, Cardenas PP, Yoshimura SH, Takeyasu K, Alonso JC. 2008. Dynamic structures of *Bacillus subtilis* RecN-DNA complexes. Nucleic Acids Res 36:110-20.
10. Taniguchi Y, Choi PJ, Li GW, Chen H, Babu M, Hearn J, Emili A, Xie XS. 2010. Quantifying *E. coli* proteome and transcriptome with single-molecule sensitivity in single cells. Science 329:533-8.
11. Skinner SO, Sepulveda LA, Xu H, Golding I. 2013. Measuring mRNA copy number in individual *Escherichia coli* cells using single-molecule fluorescent in situ hybridization. Nat Protoc 8:1100-13.
12. Cabrini G, Verkman AS. 1986. Potential-sensitive response mechanism of diS-C3-(5) in biological membranes. J Membr Biol 92:171-82.
13. Strauber H, Muller S. 2010. Viability states of bacteria--specific mechanisms of selected probes. Cytometry A 77:623-34.
14. Malik M, Capecci J, Drlica K. 2009. Lon protease is essential for paradoxical survival of *Escherichia coli* exposed to high concentrations of quinolone. Antimicrob Agents Chemother 53:3103-5.

15. Luan G, Hong Y, Drlica K, Zhao X. 2018. Suppression of reactive oxygen species accumulation accounts for paradoxical bacterial survival at high quinolone concentration. Antimicrob Agents Chemother 62:e01622-17.
16. Malik M, Hussain S, Drlica K. 2007. Effect of anaerobic growth on quinolone lethality with *Escherichia coli*. Antimicrob Agents Chemother 51:28-34.
17. Chen CR, Malik M, Snyder M, Drlica K. 1996. DNA gyrase and topoisomerase IV on the bacterial chromosome: quinolone-induced DNA cleavage. J Mol Biol 258:627-37.
18. Malik M, Zhao X, Drlica K. 2006. Lethal fragmentation of bacterial chromosomes mediated by DNA gyrase and quinolones. Mol Microbiol 61:810-25.
19. Hong Y, Li Q, Gao Q, Xie J, Huang H, Drlica K, Zhao X. 2020. Reactive oxygen species play a dominant role in all pathways of rapid quinolone-mediated killing. J Antimicrob Chemother 75:576-85.
20. Volzing KG, Brynildsen MP. 2015. Stationary-phase persisters to ofloxacin sustain DNA damage and require repair systems only during recovery. MBio 6:e00731-15.
21. Allison KR, Brynildsen MP, Collins JJ. 2011. Metabolite-enabled eradication of bacterial persisters by aminoglycosides. Nature 473:216-20.
22. Lang M, Carvalho A, Baharoglu Z, Mazel D. 2023. Aminoglycoside uptake, stress, and potentiation in Gram-negative bacteria: new therapies with old molecules. Microbiol Mol Biol Rev 87:e0003622.
23. Zeng J, Hong Y, Zhao N, Liu Q, Zhu W, Xiao L, Wang W, Chen M, Hong S, Wu L, Xue Y, Wang D, Niu J, Drlica K, Zhao X. 2022. A broadly applicable, stress-mediated bacterial death pathway regulated by the phosphotransferase system (PTS) and the cAMP-Crp cascade. Proc Natl Acad Sci U S A 119:e2118566119.
24. Hansen S, Lewis K, Vulic M. 2008. Role of global regulators and nucleotide metabolism in antibiotic tolerance in *Escherichia coli*. Antimicrob Agents Chemother 52:2718-26.
25. Nicolau SE, Lewis K. 2022. The role of integration host factor in *Escherichia coli* persister formation. mBio 13:e0342021.
26. Zheng EJ, Andrews IW, Grote AT, Manson AL, Alcantar MA, Earl AM, Collins JJ. 2022. Modulating the evolutionary trajectory of tolerance using antibiotics with different metabolic dependencies. Nat Commun 13:2525.
27. Girgis HS, Harris K, Tavazoie S. 2012. Large mutational target size for rapid emergence of bacterial persistence. Proc Natl Acad Sci U S A 109:12740-5.
